# Supplementary material for: Comparison of new psychiatric diagnoses among Finnish children and adolescents before and during the COVID-19 pandemic: A nationwide register-based study
Source: PLoS Med. 2023 Feb 27;20(2):e1004072. doi: 10.1371/journal.pmed.1004072 (PMC10089356; doi:10.1371/journal.pmed.1004072)
Supplement: S1 STROBE Checklist — (PDF) [file pmed.1004072.s001.pdf]

S1 Checklist

STROBE Statement—checklist of items that should be included in reports of observational studies

	Item No	Recommendation	Section and paragraph
**Title and abstract**	1	(*a*) Indicate the study's design with a commonly used term in the title or the abstract	Title page
		(*b*) Provide in the abstract an informative and balanced summary of what was done and what was found	Abstract page
**Introduction**			
Background/rationale	2	Explain the scientific background and rationale for the investigation being reported	Introduction section, 1-2 paragraph
Objectives	3	State specific objectives, including any prespecified hypotheses	Introduction section, last paragraph
**Methods**			
Study design	4	Present key elements of study design early in the paper	Methods section, 1st paragraph
Setting	5	Describe the setting, locations, and relevant dates, including periods of recruitment, exposure, follow-up, and data collection	Methods section, 1-3 paragraph
Participants	6	(*a*) *Cohort study*—Give the eligibility criteria, and the sources and methods of selection of participants. Describe methods of follow-up<br>*Case-control study*—Give the eligibility criteria, and the sources and methods of case ascertainment and control selection. Give the rationale for the choice of cases and controls<br>*Cross-sectional study*—Give the eligibility criteria, and the sources and methods of selection of participants	Our study was nationwide register-based study. This information is explained in 1st paragraph in the method section. The aggregated data is based on register/based data dating back to birth i.e. a cohort study
		(*b*) *Cohort study*—For matched studies, give matching criteria and number of exposed and unexposed<br>*Case-control study*—For matched studies, give matching criteria and the number of controls per case	
Variables	7	Clearly define all outcomes, exposures, predictors, potential confounders, and effect modifiers. Give diagnostic criteria, if applicable	Methods section, outcomes and stratifying variables
Data sources/ measurement	8*	For each variable of interest, give sources of data and details of methods of	Methods section, data sources

		assessment (measurement). Describe comparability of assessment methods if there is more than one group	
Bias	9	Describe any efforts to address potential sources of bias	Methods section, statistical analysis
Study size	10	Explain how the study size was arrived at	Methods section, study design, setting and time periods
Quantitative variables	11	Explain how quantitative variables were handled in the analyses. If applicable, describe which groupings were chosen and why	Methods section, outcomes and stratifying variables
Statistical methods	12	(*a*) Describe all statistical methods, including those used to control for confounding	Methods section, statistical analysis
		(*b*) Describe any methods used to examine subgroups and interactions	Methods section, statistical analysis
		(*c*) Explain how missing data were addressed	Methods section, study design, setting and time periods
		(*d*) *Cohort study*—If applicable, explain how loss to follow-up was addressed<br>*Case-control study*—If applicable, explain how matching of cases and controls was addressed<br>*Cross-sectional study*—If applicable, describe analytical methods taking account of sampling strategy	N/A
		(*e*) Describe any sensitivity analyses	

Continued on next page

**Results**			
Participants	13*	(a) Report numbers of individuals at each stage of study—eg numbers potentially eligible, examined for eligibility, confirmed eligible, included in the study, completing follow-up, and analysed	Results, description of the data
		(b) Give reasons for non-participation at each stage	
		(c) Consider use of a flow diagram	
Descriptive data	14*	(a) Give characteristics of study participants (eg demographic, clinical, social) and information on exposures and potential confounders	Results, description of the data
		(b) Indicate number of participants with missing data for each variable of interest	
		(c) *Cohort study*—Summarise follow-up time (eg, average and total amount)	
Outcome data	15*	*Cohort study*—Report numbers of outcome events or summary measures over time	Results, description of the data
		Case-control study—Report numbers in each exposure category, or summary measures of exposure	
		Cross-sectional study—Report numbers of outcome events or summary measures	
Main results	16	(*a*) Give unadjusted estimates and, if applicable, confounder-adjusted estimates and their precision (eg, 95% confidence interval). Make clear which confounders were adjusted for and why they were included	Results section, paragraphs of Diagnoses of any psychiatric or neurodevelopmental disorder and of Results by sex, age, geographic area and diagnostic groups
		(*b*) Report category boundaries when continuous variables were categorized	N/A
		(*c*) If relevant, consider translating estimates of relative risk into absolute risk for a meaningful time period	Tables 2 and 3
Other analyses	17	Report other analyses done—eg analyses of subgroups and interactions, and sensitivity analyses	Results section, last paragraph (S4 Table and S5 Figure)
**Discussion**			
Key results	18	Summarise key results with reference to study objectives	Discussion, first paragraph
Limitations	19	Discuss limitations of the study, taking into account sources of potential bias or imprecision. Discuss both direction and magnitude of any potential bias	Discussion, 8th paragraph
Interpretation	20	Give a cautious overall interpretation of results considering objectives, limitations, multiplicity of analyses, results from similar studies, and other relevant evidence	Discussion, last paragraph
Generalisability	21	Discuss the generalisability (external validity) of the study results	Discussion, 8th paragraph
**Other information**			

Funding	22	The Academy of Finland funded the study via the Special funding for research on COVID-19 epidemic and the mitigation of its effects (335690, PI Gyllenberg) and the INVEST flagship (308552, PI Sourander). The funders had no involvement in any aspect of the study or paper.	
