# Supplementary material for: Comparison of new psychiatric diagnoses among Finnish children and adolescents before and during the COVID-19 pandemic: A nationwide register-based study
Source: PLoS Med. 2023 Feb 27;20(2):e1004072. doi: 10.1371/journal.pmed.1004072 (PMC10089356; doi:10.1371/journal.pmed.1004072)
Supplement: S1 Fig — (PDF) [file pmed.1004072.s002.pdf]

S1 Fig
Weekly rate of COVID−19 patients treated in hospitals

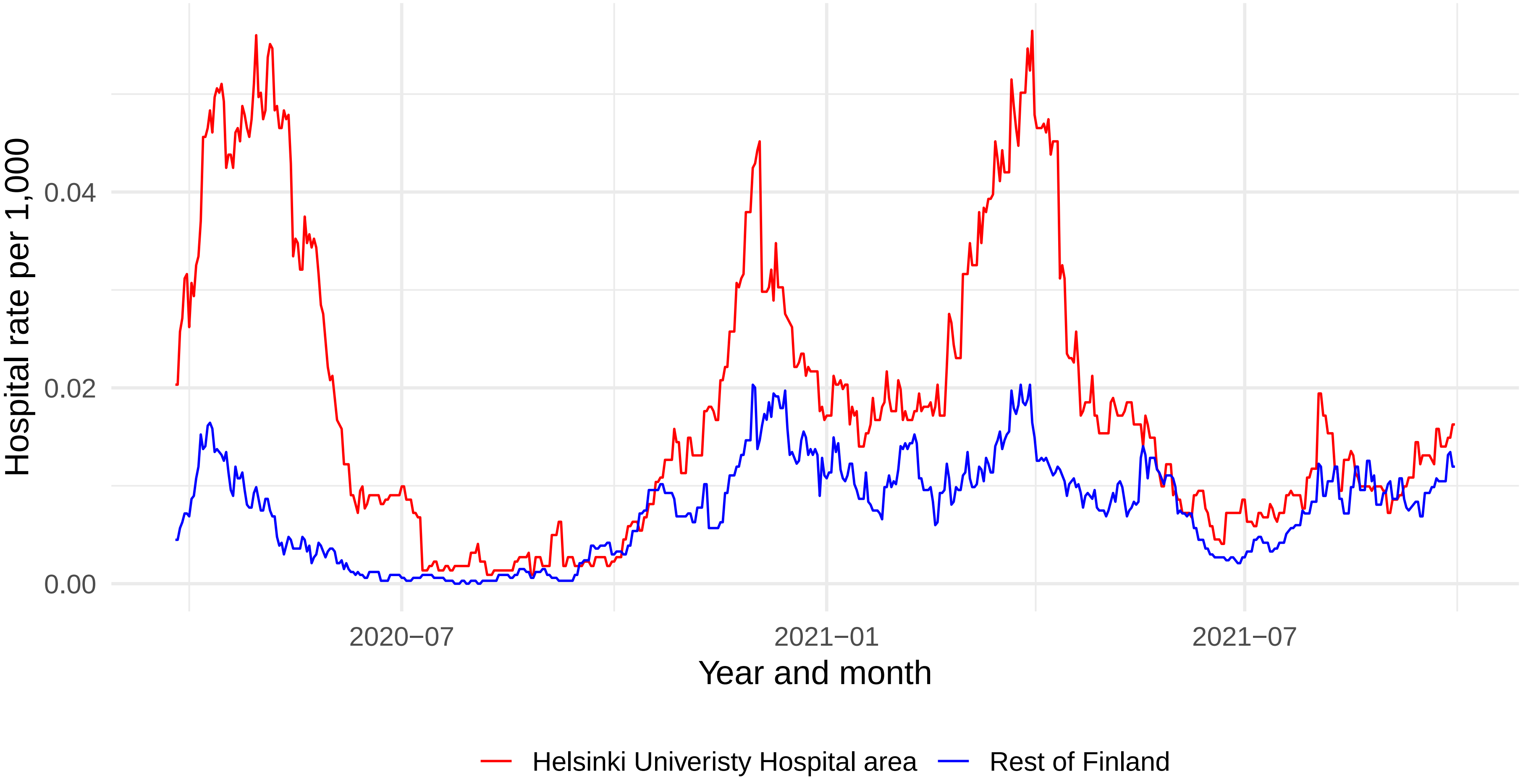

Data source: Finnish Institute for Health and Welfare
