# Supplementary figures and images for: Comparison of new psychiatric diagnoses among Finnish children and adolescents before and during the COVID-19 pandemic: A nationwide register-based study
Source: PLoS Med. 2023 Feb 27;20(2):e1004072. doi: 10.1371/journal.pmed.1004072 (PMC10089356; doi:10.1371/journal.pmed.1004072)

# S2 Fig

## Sex-stratified results for selected outcomes

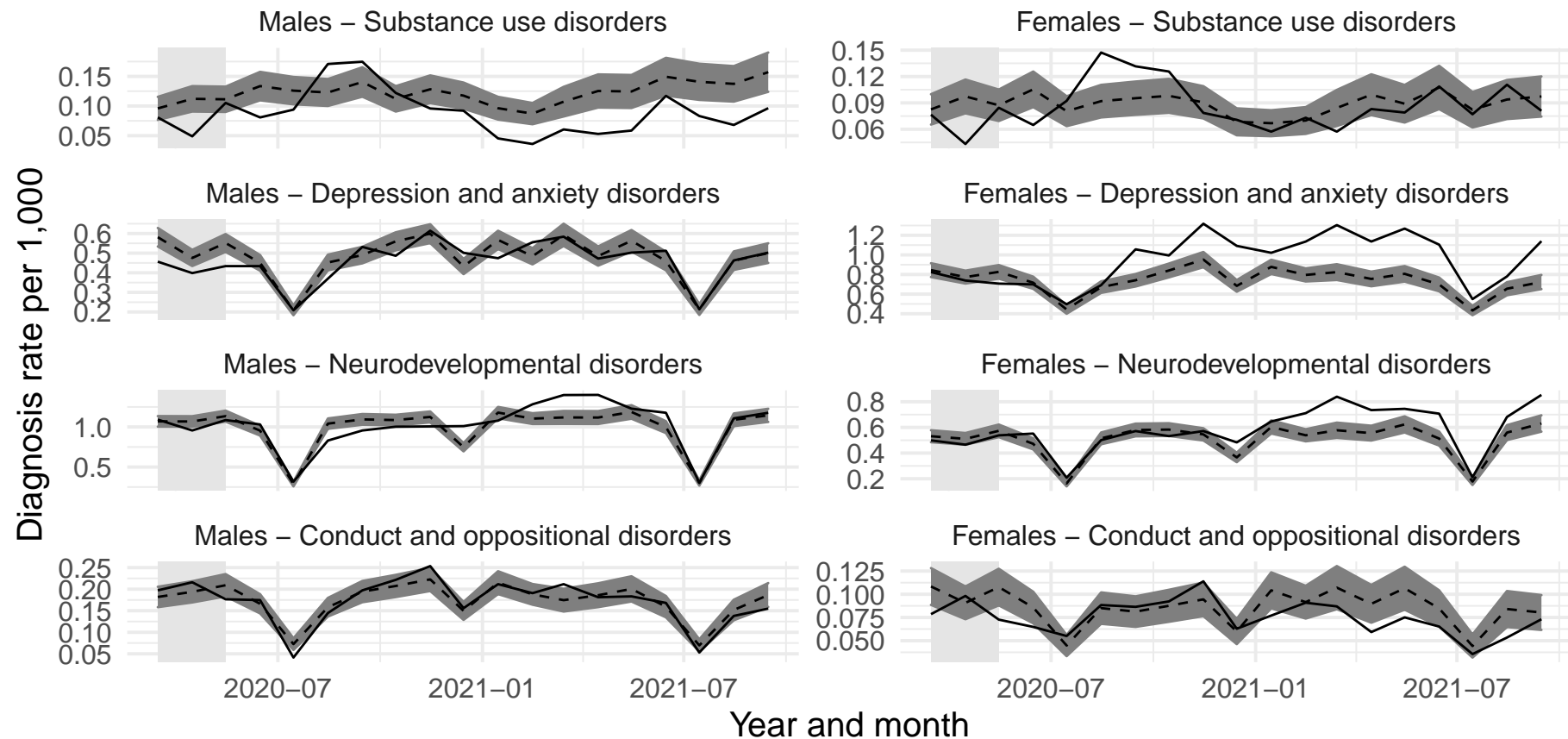

Rate — Observed - - Predicted with 95% CI

Supplement: S2 Fig — (PDF) [file pmed.1004072.s003.pdf]
