# Supplementary material for: Comparison of new psychiatric diagnoses among Finnish children and adolescents before and during the COVID-19 pandemic: A nationwide register-based study
Source: PLoS Med. 2023 Feb 27;20(2):e1004072. doi: 10.1371/journal.pmed.1004072 (PMC10089356; doi:10.1371/journal.pmed.1004072)
Supplement: S1 Supporting information — (PDF) [file pmed.1004072.s004.pdf]

S1 SUPPORTING INFORMATION

RESEARCH PLAN (2021-03-14) AND DEVIATIONS TO THE PLAN (END OF DOCUMENT)

TITLE:

Service use for psychiatric and neurodevelopmental disorders among children and adolescents before and during the COVID-19-pandemic

SIGNIFICANCE

There are initial indicators that mental health has worsened among children and adolescents after the COVID-pandemic [1, 2]. If this is true, it can have long-going consequences, as mental health problems during development predicts a wide range of long-term adverse events [3, 4]. Further, there are reports from the UK of decreases in mental health service use in the beginning of the COVID-19-pandemic [5-7], but longer follow-up studies are lacking. A major challenge remains how services for psychiatric and neurodevelopmental disorders should be tailored during and following the COVID-19-pandemic, so that the risk of mental health problems can be minimized. In order to achieve that goal, timely information on service use changes for psychiatric and neurodevelopmental disorders among children and adolescents before and during the COVID-19-pandemic is needed.

SPECIFIC AIMS

The hypothesis is that incidence service use for psychiatric and neurodevelopmental disorders decreased when the COVID-19-lockdown started and that the incidence service use increased from August 2020, indicating a delayed treatment of unmet need following the lock-down. The specific aims are:

1. To compare the monthly-specific incidence rates of service use for psychiatric and neurodevelopmental disorders in 2020-2021 to the corresponding rates in 2017–2019, and test whether the relative changes decreased in March-July and increased after August 2020.
2. To describe the relative change of monthly-specific incidence rates for specific disorders in specialized services and for hospital-presenting self-harm.
3. To test whether decreased relative changes of monthly-specific incidence rates for any disorder in March-July is associated with increased relative changes for hospital-presenting self-harm among adolescents in after August 2020.

METHODS

The study is a register-based study with individual-level data retrieved internally at THL further visualized and analyzed at the aggregated level. The study population is all children and adolescents aged 0-17 years living in Finland between January 1, 2017, and December 31, 2021. As the study is register-based, no informed consent by the participants according to Finnish law. The study seeks approval by THL.

Data sources

Study population:
The number of subjects aged 0-17 years in 2017–2021 is retrieved from publicly available census information at Statistics Finland. The number of subjects will be based on the latest census at December 31 for available years (years 2017-2019 at 2021-0304) and the official projected census for other years (years 2020-2021 at 2021-03-04). The monthly-specific number of subjects will be linearly interpolated from the available yearly-specific data at

December 31. All numbers will be stratified by gender, age group (0-12 and 13-17 years) and university central hospital specific catchment areas (ER1 Helsinki, ER2 Turku, ER3 Tampere, ER4 Kuopio, ER5 Oulu and ER6 Åland).

Incident service use:
The monthly-specific number of subjects with incident specialized service use for psychiatric and neurodevelopmental disorders and self-harm is based on the Care Register for Health Care (HILMO) and Primary Care Register (AvoHILMO). The Care Register for Health Care register contain information on inpatient care and specialized service use in public outpatient clinics; the outpatient data is available since 1998, i.e., for the whole lifetime in the study population. The Primary Care Register is available since 2011, but comprehensive only later years. The incident service use for specialized service use will take into consideration lifetime service use with the studied diagnosis, while incident service use for primary care will take into account a two-year washout-period due to lack of lifetime data. In the specialized service use data, no restrictions to medical specialties will be made, and both primary and any secondary diagnoses and diagnoses for external causes will be taken into account. All monthly-specific number of subjects will be retrieved separately for diagnostic groups and special outcomes of interest (inpatient care, intentional self-harm) as described in Table 1 and partially based on our previous work [8-10]. We will focus on eleven main outcomes and retrieve information on 28 more specific secondary outcomes for additional analyses (Table 1). The numbers of subjects in the aggregated data will be stratified by gender, age group (0-12 and 13-17 years) and official university central hospital specific catchment areas (ER1 Helsinki, ER2 Turku, ER3 Tampere, ER4 Kuopio, ER5 Oulu and ER6 Åland).

Data management and data security:
The study population data will be retrieved by downloading it from publicly available data. The data on the outcome, incident service use, will be summarized internally at THL with in-house high standards of data security. The summarized data on the outcome will be on the aggregated level and will not contain any individual data; instead, we will operate with a matrix containing the number of incident cases stratified by month (n=5*12=60), diagnostic group (Table 1; n=39), gender (n=2), age group (n=2) and university central hospital specific catchment areas (n=6), resulting in a total of 56,160 cells (60*39*2*2*6). Following review of the aggregated data in-house at THL by the team's data manager (Ms. Liukko), the aggregated data will be made available to the study group.

Descriptive analyses and statistical modelling:
The primary descriptions of the outcomes will be relative monthly-specific change of the studied diagnoses during the months of 2020-2021 compared to the corresponding months in 2017-2019. This will enable us to take into account generally occurring seasonal variation of service use. To test for the hypothesis in specific aim 1 (decrease in use during lock-down-months followed by an increase), we will model the count data with generalized linear models with Poisson distribution. Specific aim number 2 is descriptive, and for that purpose, we will rely on data visualization techniques. To test for the hypothesis in specific aim 3 (decrease in service use during lock-down is associated with later increased hospital-presenting self-harm), we will restrict to adolescents (age 13-17) among which self-harm is more common, and test for correlation between A) the relative monthly specific change of any service use during March-July and B) the lagged relative monthly specific change of hospital presenting self-harm during August-December; the observations for the analyses will be the sociodemographic strata (gender, area) and we will weigh the analyses for population size. Further panel data analyses will be performed as appropriate.

Sensitivity analyses:
Given that the rate of covid-infections and the economic consequences have varied in different areas, we will collect publicly monthly available data on covid-19-infections and

increased unemployment for different areas. Then, we will divide the geographic areas to those with high rates of infection and highly increased employment versus areas without lower rates and less increased employment, and conduct the main analyses stratified by these characteristics. Further, as a new electronic health record system was taken into use in the Helsinki university central hospital specific catchment area during 2020 – something which can affect the validity of relative changes in registered service use – we will stratify the main analyses with the categorization Helsinki university central hospital specific catchment area vs. rest of Finland.

Open data principles:
In line with the funder's principles (Academy of Finland), the team follows an open data strategy to promote transparency, reproducibility and replication of the scientific process, but restricting data sharing in case of sensitive material, described in detail as follows. This protocol and the code to perform the analyses will be made publicly available at time of submission of a preprint or publication of the scientific report. The scientific report will be made open access. The aggregated data will be made publicly available at time of submission of a preprint or publication of the scientific report with the following restrictions: if a time-series on a specific diagnosis in a specific stratum contains cell count ≤5, the time-series will not be made publicly available at the stratified level to minimize the risk of individual recognition (e.g. incident self-harm during some month in some specific area); for such stratified time-series, the time-series will be made available for larger aggregations, e.g. whole Finland and not specific areas.

IMPLICATIONS AND DISSEMINATION

The findings will have implications how services for psychiatric and neurodevelopmental disorders among youth should be organized to mitigate the consequences of the COVID-19-pandemic. The study will be resulting in a scientific paper that will be published in an international peer-reviewed journal. Further, we will disseminate the findings to stakeholders and the wider public by participating in events related to COVID-19-related organizational changes, and by describing the results in non-technical terms in social media.

DEVIATIONS TO THE ANALYTIC PLAN BY 2022-05-27 AND RATIONALE FOR THE DEVIATIONS

- In specific aims, we tested the months differently
    - The original assumption was "decreased in March-July 2020"
    - After inspecting the data, the summer months June and July 2020 had low counts of incidence cases, and a later published systematic review [11] showed that service use was in particular affected during the spring months in 2020.
    - During the writing process we gained access to follow-up until September 2021
    - We did not test the third hypothesis about increased hospital presenting self-harm, because it decreased in the primary analyses
- The definition of geographic area was simplified from six areas to comparing Helsinki University Central Hospital area to the rest of Finland, as the number of COVID-19-cases were particularly high in the Helsinki area
- We did not include new primary care diagnoses, because we could not assure that they were new diagnoses due to the considerably shorter follow-up time.
- We used negative binomial models instead of Poisson models to fit the data due to the variance in the data. In additional analyses with Poisson models, the results remained similar.

REFERENCES

1. Newlove-Delgado, T., McManus, S., Sadler, K., Thandi, S., Vizard, T., Cartwright, C., & Ford, T. (2021). Child mental health in England before and during the COVID-19 lockdown. The Lancet Psychiatry. doi:10.1016/S2215-0366(20)30570-8
2. Tanaka, T., & Okamoto, S. (2021). Increase in suicide following an initial decline during the COVID-19 pandemic in Japan. Nat Hum Behav. doi:10.1038/s41562-020-01042-z
3. Gyllenberg, D., Sourander, A., Niemel√§, S., Helenius, H., Sillanm√§ki, L., Ristkari, T., . . . Almqvist, F. (2011). Childhood predictors of use and costs of antidepressant medication by age 24 years: findings from the finnish nationwide 1981 birth cohort study. Journal of the American Academy of Child and Adolescent Psychiatry, 50(4), 406-415.e401. doi:10.1016/j.jaac.2010.12.016
4. Sourander, A., Gyllenberg, D., Brunstein Klomek, A., Sillanmaki, L., Ilola, A. M., & Kumpulainen, K. (2016). Association of Bullying Behavior at 8 Years of Age and Use of Specialized Services for Psychiatric Disorders by 29 Years of Age. JAMA psychiatry, 73(2), 159-165. doi:10.1001/jamapsychiatry.2015.2419
5. Chen, S., She, R., Qin, P., Kershenbaum, A., Fernandez-Egea, E., Nelder, J. R., . . . Cardinal, R. N. (2020). The Medium-Term Impact of COVID-19 Lockdown on Referrals to Secondary Care Mental Health Services: A Controlled Interrupted Time Series Study. Frontiers in psychiatry, 11, 585915. doi:10.3389/fpsyt.2020.585915
6. Mansfield, K. E., Mathur, R., Tazare, J., Henderson, A. D., Mulick, A. R., Carreira, H., . . . Langan, S. M. (2021). Indirect acute effects of the COVID-19 pandemic on physical and mental health in the UK: a population-based study. Lancet Digit Health. doi:10.1016/S2589-7500(21)00017-0
7. Williams, R., Jenkins, D. A., Ashcroft, D. M., Brown, B., Campbell, S., Carr, M. J., . . . Peek, N. (2020). Diagnosis of physical and mental health conditions in primary care during the COVID-19 pandemic: a retrospective cohort study. Lancet Public Health, 5(10), e543-e550. doi:10.1016/S2468-2667(20)30201-2
8. Gyllenberg, D., Gissler, M., Malm, H., Artama, M., Hinkka-Yli-Salomaki, S., Brown, A. S., & Sourander, A. (2014). Specialized service use for psychiatric and neurodevelopmental disorders by age 14 in Finland. Psychiatric services, 65(3), 367-373. doi:10.1176/appi.ps.201200544
9. Gyllenberg, D., Marttila, M., Sund, R., Jokiranta-Olkoniemi, E., Sourander, A., Gissler, M., & Ristikari, T. (2018). Temporal changes in the incidence of treated psychiatric and neurodevelopmental disorders during adolescence: an analysis of two national Finnish birth cohorts. Lancet Psychiatry, 5(3), 227-236. doi:10.1016/S2215-0366(18)30038-5
10. Kerkela, M., Gyllenberg, D., Gissler, M., Sillanmaki, L., Keski-Santti, M., Hinkka-Yli-Salomaki, S., . . . Veijola, J. (2021). Cumulative incidences of hospital-treated psychiatric disorders are increasing in five Finnish birth cohorts. Acta psychiatrica Scandinavica, 143(2), 119-129. doi:10.1111/acps.13247
11. Wan Mohd Yunus WMA, Kauhanen L, Sourander A, Brown JSL, Peltonen K, Mishina K, Lempinen L, Bastola K, Gilbert S, Gyllenberg D. Registered psychiatric service use, self-harm and suicides of children and young people aged 0-24 before and during the COVID-19 pandemic: a systematic review. Child Adolesc Psychiatry Ment Health. 2022 Feb 25;16(1):15. doi: 10.1186/s13034-022-00452-3
