# Supplementary material for: Comparison of new psychiatric diagnoses among Finnish children and adolescents before and during the COVID-19 pandemic: A nationwide register-based study
Source: PLoS Med. 2023 Feb 27;20(2):e1004072. doi: 10.1371/journal.pmed.1004072 (PMC10089356; doi:10.1371/journal.pmed.1004072)
Supplement: S2 Supporting information — (PDF) [file pmed.1004072.s005.pdf]

S2 Supporting information

Tables showing the observed and predicted number of diagnoses by month during the COVID-19 pandemic in Finland until September 2021.

Table A. Any diagnosis

Year	Month	Observed	Predicted No.	Absolute difference No.	Relative difference %
2020	3	1465	1433 (1356 – 1510)	32 (-45 – 109)	2.3 (-3 – 8.1)
2020	4	1311	1386 (1311 – 1461)	-75 (-150 – 0)	-5.4 (-10.3 – 0)
2020	5	1355	1501 (1421 – 1582)	-146 (-227 – -66)	-9.8 (-14.3 – -4.7)
2020	6	1331	1348 (1275 – 1421)	-17 (-90 – 56)	-1.3 (-6.4 – 4.4)
2020	7	682	629 (590 – 669)	53 (13 – 92)	8.4 (1.9 – 15.7)
2020	8	1233	1316 (1244 – 1388)	-83 (-155 – -11)	-6.3 (-11.1 – -0.9)
2020	9	1519	1373 (1299 – 1447)	146 (72 – 220)	10.6 (4.9 – 17)
2020	10	1528	1458 (1380 – 1537)	70 (-9 – 148)	4.8 (-0.6 – 10.7)
2020	11	1644	1556 (1473 – 1639)	88 (5 – 171)	5.7 (0.3 – 11.6)
2020	12	1548	1102 (1040 – 1164)	446 (384 – 508)	40.5 (33 – 48.8)
2021	1	1680	1538 (1456 – 1620)	142 (60 – 224)	9.2 (3.7 – 15.4)
2021	2	1881	1372 (1298 – 1446)	509 (435 – 583)	37.1 (30.1 – 44.9)
2021	3	2067	1422 (1333 – 1511)	645 (556 – 734)	45.4 (36.8 – 55.1)
2021	4	1870	1376 (1289 – 1462)	494 (408 – 581)	35.9 (27.9 – 45.1)
2021	5	1785	1490 (1397 – 1583)	295 (202 – 388)	19.8 (12.7 – 27.8)
2021	6	1735	1338 (1253 – 1423)	397 (312 – 482)	29.7 (21.9 – 38.4)
2021	7	710	625 (580 – 669)	85 (41 – 130)	13.6 (6.1 – 22.4)
2021	8	1487	1306 (1223 – 1389)	181 (98 – 264)	13.9 (7.1 – 21.6)
2021	9	1733	1363 (1277 – 1449)	370 (284 – 456)	27.2 (19.6 – 35.7)

Table B. Any diagnosis by sex

Sex	Year	Month	Observed	Predicted No.	Absolute difference No.	Relative difference %
Males	2020	3	777	778 (737 – 818)	-1 (-41 – 40)	-0.1 (-5 – 5.4)
Males	2020	4	666	755 (715 – 795)	-89 (-129 – -49)	-11.8 (-16.2 – -6.9)
Males	2020	5	748	830 (787 – 872)	-82 (-124 – -39)	-9.8 (-14.2 – -5)
Males	2020	6	724	731 (692 – 769)	-7 (-45 – 32)	-0.9 (-5.9 – 4.7)
Males	2020	7	306	297 (275 – 319)	9 (-13 – 31)	3.1 (-4 – 11.2)
Males	2020	8	604	746 (707 – 786)	-142 (-182 – -103)	-19.1 (-23.1 – -14.5)
Males	2020	9	741	761 (721 – 801)	-20 (-60 – 20)	-2.6 (-7.5 – 2.8)
Males	2020	10	755	775 (734 – 815)	-20 (-60 – 21)	-2.6 (-7.4 – 2.8)
Males	2020	11	744	852 (808 – 895)	-108 (-151 – -64)	-12.6 (-16.9 – -8)
Males	2020	12	770	595 (561 – 629)	175 (141 – 209)	29.5 (22.5 – 37.3)
Males	2021	1	832	828 (785 – 870)	4 (-38 – 47)	0.5 (-4.4 – 5.9)
Males	2021	2	956	738 (699 – 777)	218 (179 – 257)	29.5 (23.1 – 36.7)
Males	2021	3	1011	780 (732 – 827)	231 (184 – 279)	29.7 (22.2 – 38.1)
Males	2021	4	930	757 (710 – 803)	173 (127 – 220)	22.9 (15.8 – 31)
Males	2021	5	830	832 (782 – 882)	-2 (-52 – 48)	-0.2 (-5.9 – 6.2)
Males	2021	6	836	732 (687 – 778)	104 (58 – 149)	14.1 (7.5 – 21.7)
Males	2021	7	310	298 (274 – 321)	12 (-11 – 36)	4.2 (-3.6 – 13.2)
Males	2021	8	766	748 (702 – 794)	18 (-28 – 64)	2.4 (-3.6 – 9.1)
Males	2021	9	819	763 (716 – 810)	56 (9 – 103)	7.4 (1.1 – 14.4)
Females	2020	3	688	654 (605 – 703)	34 (-15 – 83)	5.2 (-2.1 – 13.6)
Females	2020	4	645	630 (583 – 678)	15 (-33 – 62)	2.3 (-4.8 – 10.6)
Females	2020	5	607	671 (622 – 721)	-64 (-114 – -15)	-9.6 (-15.8 – -2.4)
Females	2020	6	607	617 (570 – 663)	-10 (-56 – 37)	-1.6 (-8.5 – 6.4)
Females	2020	7	376	330 (302 – 359)	46 (17 – 74)	13.9 (4.8 – 24.6)
Females	2020	8	629	570 (527 – 614)	59 (15 – 102)	10.3 (2.4 – 19.4)
Females	2020	9	778	612 (566 – 658)	166 (120 – 212)	27.1 (18.2 – 37.5)
Females	2020	10	773	682 (631 – 732)	91 (41 – 142)	13.3 (5.5 – 22.4)
Females	2020	11	900	704 (652 – 755)	196 (145 – 248)	27.9 (19.1 – 38.1)
Females	2020	12	778	507 (467 – 546)	271 (232 – 311)	53.6 (42.4 – 66.6)
Females	2021	1	848	709 (657 – 761)	139 (87 – 191)	19.7 (11.5 – 29.1)
Females	2021	2	925	632 (585 – 679)	293 (246 – 340)	46.3 (36.1 – 58)
Females	2021	3	1056	641 (586 – 697)	415 (359 – 470)	64.6 (51.4 – 80.3)
Females	2021	4	940	618 (564 – 672)	322 (268 – 376)	52.1 (39.8 – 66.6)
Females	2021	5	955	658 (601 – 716)	297 (239 – 354)	45.1 (33.5 – 58.8)
Females	2021	6	899	605 (552 – 658)	294 (241 – 347)	48.6 (36.7 – 63)
Females	2021	7	400	324 (292 – 355)	76 (45 – 108)	23.5 (12.6 – 36.8)
Females	2021	8	721	559 (510 – 609)	162 (112 – 211)	28.9 (18.4 – 41.5)
Females	2021	9	914	600 (547 – 653)	314 (261 – 367)	52.3 (40 – 67)

Table C. Any diagnosis by age group

Age	Year	Month	Observed	Predicted No.	Absolute difference No.	Relative difference %
0 - 12 years	2020	3	931	898 (846 – 949)	33 (-18 – 85)	3.7 (-1.9 – 10)
0 - 12 years	2020	4	854	879 (828 – 930)	-25 (-76 – 26)	-2.9 (-8.2 – 3.1)
0 - 12 years	2020	5	882	955 (901 – 1010)	-73 (-128 – -19)	-7.7 (-12.6 – -2.1)
0 - 12 years	2020	6	899	871 (821 – 922)	28 (-23 – 78)	3.2 (-2.5 – 9.5)
0 - 12 years	2020	7	374	318 (293 – 342)	56 (32 – 81)	17.7 (9.3 – 27.5)
0 - 12 years	2020	8	739	907 (855 – 959)	-168 (-220 – -116)	-18.5 (-23 – -13.6)
0 - 12 years	2020	9	861	946 (892 – 999)	-85 (-138 – -31)	-9 (-13.8 – -3.5)
0 - 12 years	2020	10	935	958 (904 – 1013)	-23 (-78 – 31)	-2.4 (-7.7 – 3.4)
0 - 12 years	2020	11	937	1001 (945 – 1057)	-64 (-120 – -8)	-6.4 (-11.3 – -0.8)
0 - 12 years	2020	12	903	696 (654 – 739)	207 (164 – 249)	29.7 (22.2 – 38.1)
0 - 12 years	2021	1	1007	972 (917 – 1027)	35 (-20 – 90)	3.6 (-2 – 9.8)
0 - 12 years	2021	2	1132	885 (835 – 936)	247 (196 – 297)	27.9 (20.9 – 35.6)
0 - 12 years	2021	3	1250	905 (844 – 966)	345 (284 – 406)	38.1 (29.4 – 48)
0 - 12 years	2021	4	1148	886 (827 – 946)	262 (202 – 321)	29.5 (21.4 – 38.9)
0 - 12 years	2021	5	1062	963 (899 – 1027)	99 (35 – 163)	10.2 (3.4 – 18.1)
0 - 12 years	2021	6	1052	878 (819 – 938)	174 (114 – 233)	19.8 (12.2 – 28.4)
0 - 12 years	2021	7	363	320 (293 – 347)	43 (16 – 70)	13.3 (4.5 – 23.8)
0 - 12 years	2021	8	965	915 (853 – 976)	50 (-11 – 112)	5.5 (-1.1 – 13.1)
0 - 12 years	2021	9	1055	953 (890 – 1017)	102 (38 – 165)	10.7 (3.8 – 18.5)
13 - 17 years	2020	3	534	534 (498 – 570)	0 (-36 – 36)	0 (-6.3 – 7.2)
13 - 17 years	2020	4	457	506 (472 – 541)	-49 (-84 – -15)	-9.8 (-15.5 – -3.2)
13 - 17 years	2020	5	473	546 (509 – 583)	-73 (-110 – -36)	-13.4 (-18.8 – -7.1)
13 - 17 years	2020	6	432	478 (445 – 512)	-46 (-80 – -13)	-9.7 (-15.6 – -3)
13 - 17 years	2020	7	308	305 (281 – 329)	3 (-21 – 27)	0.9 (-6.4 – 9.6)
13 - 17 years	2020	8	494	414 (385 – 444)	80 (50 – 109)	19.2 (11.2 – 28.5)
13 - 17 years	2020	9	658	433 (402 – 464)	225 (194 – 256)	51.9 (41.8 – 63.5)
13 - 17 years	2020	10	593	502 (468 – 537)	91 (56 – 125)	18 (10.5 – 26.7)
13 - 17 years	2020	11	707	556 (519 – 593)	151 (114 – 188)	27.1 (19.1 – 36.2)
13 - 17 years	2020	12	645	405 (376 – 435)	240 (210 – 269)	59.1 (48.3 – 71.5)
13 - 17 years	2021	1	673	564 (527 – 602)	109 (71 – 146)	19.3 (11.8 – 27.8)
13 - 17 years	2021	2	749	487 (454 – 520)	262 (229 – 295)	53.8 (44 – 65.1)
13 - 17 years	2021	3	817	516 (475 – 557)	301 (260 – 342)	58.3 (46.7 – 71.9)
13 - 17 years	2021	4	722	489 (450 – 528)	233 (194 – 272)	47.5 (36.6 – 60.3)
13 - 17 years	2021	5	723	528 (486 – 569)	195 (154 – 237)	37 (27 – 48.8)
13 - 17 years	2021	6	683	462 (425 – 500)	221 (183 – 258)	47.8 (36.7 – 60.7)
13 - 17 years	2021	7	347	295 (269 – 321)	52 (26 – 78)	17.7 (8.1 – 29.2)
13 - 17 years	2021	8	522	400 (367 – 434)	122 (88 – 155)	30.4 (20.4 – 42.2)
13 - 17 years	2021	9	678	419 (384 – 453)	259 (225 – 294)	62 (49.6 – 76.5)

Table D. Any diagnosis in Helsinki University Hospital area

Year	Month	Observed	Predicted No.	Absolute difference No.	Relative difference %
2020	3	609	618 (578 – 658)	-9 (-49 – 31)	-1.4 (-7.4 – 5.3)
2020	4	601	578 (540 – 616)	23 (-15 – 61)	4 (-2.4 – 11.3)
2020	5	588	634 (594 – 675)	-46 (-87 – -6)	-7.3 (-12.9 – -1)
2020	6	590	553 (517 – 590)	37 (0 – 73)	6.6 (0 – 14.2)
2020	7	326	272 (249 – 294)	54 (32 – 77)	20 (10.9 – 30.8)
2020	8	483	552 (516 – 589)	-69 (-106 – -33)	-12.5 (-17.9 – -6.3)
2020	9	587	562 (525 – 599)	25 (-12 – 62)	4.5 (-2 – 11.8)
2020	10	575	582 (544 – 620)	-7 (-45 – 31)	-1.3 (-7.3 – 5.6)
2020	11	637	626 (586 – 666)	11 (-29 – 51)	1.7 (-4.4 – 8.7)
2020	12	658	474 (441 – 506)	184 (152 – 217)	38.9 (29.9 – 49.2)
2021	1	865	649 (607 – 690)	216 (175 – 258)	33.4 (25.4 – 42.4)
2021	2	951	562 (525 – 599)	389 (352 – 426)	69.2 (58.8 – 81.1)
2021	3	1068	613 (567 – 659)	455 (409 – 501)	74.2 (62 – 88.4)
2021	4	946	574 (530 – 617)	372 (329 – 416)	64.9 (53.2 – 78.6)
2021	5	841	629 (582 – 677)	212 (164 – 259)	33.6 (24.3 – 44.5)
2021	6	806	549 (507 – 591)	257 (215 – 299)	46.8 (36.3 – 59.1)
2021	7	304	270 (245 – 294)	34 (10 – 59)	12.8 (3.4 – 24.1)
2021	8	667	548 (506 – 590)	119 (77 – 161)	21.8 (13 – 31.9)
2021	9	829	558 (515 – 600)	271 (229 – 314)	48.7 (38.1 – 61.1)

Table E. Any diagnosis in rest of Finland

Year	Month	Observed	Predicted No.	Absolute difference No.	Relative difference %
2020	3	856	814 (763 – 865)	42 (-9 – 93)	5.1 (-1 – 12.1)
2020	4	710	807 (757 – 857)	-97 (-147 – -47)	-12 (-17.2 – -6.2)
2020	5	767	866 (813 – 920)	-99 (-153 – -46)	-11.5 (-16.6 – -5.7)
2020	6	741	794 (744 – 844)	-53 (-103 – -3)	-6.7 (-12.2 – -0.5)
2020	7	356	357 (330 – 385)	-1 (-29 – 26)	-0.4 (-7.5 – 7.8)
2020	8	750	763 (715 – 811)	-13 (-61 – 35)	-1.7 (-7.5 – 4.9)
2020	9	932	810 (760 – 861)	122 (71 – 172)	15 (8.3 – 22.7)
2020	10	953	875 (821 – 929)	78 (24 – 132)	8.9 (2.6 – 16.1)
2020	11	1007	929 (872 – 985)	78 (22 – 135)	8.4 (2.2 – 15.5)
2020	12	890	628 (586 – 669)	262 (221 – 304)	41.8 (33 – 51.8)
2021	1	815	888 (834 – 942)	-73 (-127 – -19)	-8.2 (-13.5 – -2.3)
2021	2	930	809 (759 – 859)	121 (71 – 171)	15 (8.3 – 22.6)
2021	3	999	808 (749 – 866)	191 (133 – 250)	23.7 (15.3 – 33.4)
2021	4	924	801 (742 – 859)	123 (65 – 182)	15.4 (7.6 – 24.5)
2021	5	944	859 (797 – 921)	85 (23 – 147)	9.8 (2.5 – 18.4)
2021	6	929	788 (730 – 845)	141 (84 – 199)	17.9 (9.9 – 27.2)
2021	7	406	355 (324 – 385)	51 (21 – 82)	14.5 (5.5 – 25.1)
2021	8	820	757 (701 – 812)	63 (8 – 119)	8.3 (0.9 – 16.9)
2021	9	904	804 (745 – 862)	100 (42 – 159)	12.4 (4.8 – 21.3)

Table F. Substance use disorders

Year	Month	Observed	Predicted No.	Absolute difference No.	Relative difference %
2020	3	82	93 (80 – 107)	-11 (-25 – 2)	-12.2 (-23.3 – 2.8)
2020	4	48	110 (95 – 125)	-62 (-77 – -47)	-56.2 (-61.5 – -49.2)
2020	5	99	104 (89 – 118)	-5 (-19 – 10)	-4.4 (-16.1 – 11.2)
2020	6	76	125 (108 – 141)	-49 (-65 – -32)	-39 (-46.1 – -29.8)
2020	7	97	107 (92 – 122)	-10 (-25 – 5)	-9.3 (-20.3 – 5.3)
2020	8	166	112 (97 – 127)	54 (39 – 69)	48.5 (30.7 – 71.9)
2020	9	160	122 (106 – 138)	38 (22 – 54)	30.8 (15.5 – 50.7)
2020	10	129	110 (95 – 125)	19 (4 – 34)	17.4 (3.2 – 36)
2020	11	91	114 (98 – 129)	-23 (-38 – -7)	-20 (-29.5 – -7.4)
2020	12	85	96 (82 – 110)	-11 (-25 – 3)	-11.5 (-22.6 – 3.4)
2021	1	53	84 (72 – 97)	-31 (-44 – -19)	-37.1 (-45.2 – -26.1)
2021	2	56	82 (69 – 94)	-26 (-38 – -13)	-31.4 (-40.3 – -19.3)
2021	3	61	99 (83 – 116)	-38 (-55 – -22)	-38.7 (-47.5 – -26.3)
2021	4	70	117 (98 – 136)	-47 (-66 – -28)	-40 (-48.3 – -28.5)
2021	5	71	110 (92 – 128)	-39 (-57 – -21)	-35.6 (-44.7 – -23.1)
2021	6	117	133 (112 – 153)	-16 (-36 – 5)	-11.9 (-23.8 – 4.4)
2021	7	83	114 (95 – 132)	-31 (-49 – -12)	-27.1 (-37.3 – -13)
2021	8	92	119 (100 – 138)	-27 (-46 – -8)	-22.8 (-33.4 – -8)
2021	9	92	130 (110 – 151)	-38 (-59 – -18)	-29.4 (-39 – -16.3)

Table G. Psychotic and bipolar disorders

Year	Month	Observed	Predicted No.	Absolute difference No.	Relative difference %
2020	3	11	25 (19 – 31)	-14 (-20 – -8)	-56.1 (-65 – -40.9)
2020	4	26	22 (16 – 28)	4 (-2 – 10)	18.3 (-6.8 – 61.7)
2020	5	22	25 (19 – 32)	-3 (-10 – 3)	-13.2 (-30.8 – 16.5)
2020	6	29	22 (16 – 28)	7 (1 – 13)	30.1 (2.7 – 77.5)
2020	7	18	19 (13 – 24)	-1 (-6 – 5)	-3.4 (-24.8 – 35.3)
2020	8	37	26 (19 – 32)	11 (5 – 18)	44.3 (15 – 93.4)
2020	9	33	29 (22 – 36)	4 (-3 – 11)	12.6 (-9.4 – 48.6)
2020	10	41	36 (28 – 44)	5 (-3 – 13)	14.8 (-6.5 – 48.5)
2020	11	34	33 (25 – 40)	1 (-6 – 9)	4.1 (-15.7 – 35.8)
2020	12	27	29 (22 – 36)	-2 (-9 – 5)	-5.9 (-24.4 – 24.5)
2021	1	31	29 (22 – 36)	2 (-5 – 9)	7.1 (-13.9 – 41.6)
2021	2	14	19 (13 – 24)	-5 (-10 – 1)	-24.8 (-41.1 – 3.8)
2021	3	33	24 (17 – 31)	9 (2 – 16)	37.4 (5.9 – 95.6)
2021	4	19	21 (15 – 28)	-2 (-9 – 4)	-9.9 (-31.1 – 30.3)
2021	5	33	24 (17 – 32)	9 (1 – 16)	35.7 (4.7 – 93)
2021	6	20	21 (15 – 28)	-1 (-8 – 5)	-6.5 (-28.5 – 35)
2021	7	18	18 (12 – 24)	0 (-6 – 6)	0.7 (-23.9 – 49)
2021	8	26	25 (17 – 32)	1 (-6 – 9)	5.7 (-18.5 – 50.1)
2021	9	33	28 (20 – 36)	5 (-3 – 13)	17.4 (-8.7 – 64.4)

Table H. Depression and anxiety disorders

Year	Month	Observed	Predicted No.	Absolute difference No.	Relative difference %
2020	3	663	738 (685 – 791)	-75 (-128 – -22)	-10.2 (-16.2 – -3.2)
2020	4	588	647 (600 – 695)	-59 (-107 – -12)	-9.1 (-15.3 – -1.9)
2020	5	591	714 (662 – 765)	-123 (-174 – -71)	-17.2 (-22.8 – -10.7)
2020	6	587	604 (559 – 649)	-17 (-62 – 28)	-2.8 (-9.6 – 5)
2020	7	364	339 (310 – 367)	25 (-3 – 54)	7.5 (-0.9 – 17.4)
2020	8	549	582 (538 – 625)	-33 (-76 – 11)	-5.6 (-12.2 – 2)
2020	9	821	638 (591 – 685)	183 (136 – 230)	28.8 (19.9 – 39)
2020	10	765	726 (673 – 778)	39 (-13 – 92)	5.4 (-1.6 – 13.6)
2020	11	998	804 (747 – 861)	194 (137 – 251)	24.2 (16 – 33.7)
2020	12	823	578 (535 – 621)	245 (202 – 288)	42.4 (32.5 – 54)
2021	1	768	744 (691 – 798)	24 (-30 – 77)	3.2 (-3.7 – 11.1)
2021	2	869	660 (612 – 707)	209 (162 – 257)	31.8 (22.8 – 42.1)
2021	3	969	728 (667 – 790)	241 (179 – 302)	33 (22.7 – 45.2)
2021	4	825	639 (584 – 693)	186 (132 – 241)	29.2 (19 – 41.3)
2021	5	908	704 (645 – 764)	204 (144 – 263)	28.9 (18.9 – 40.8)
2021	6	830	596 (545 – 648)	234 (182 – 285)	39.2 (28.1 – 52.3)
2021	7	391	334 (302 – 366)	57 (25 – 89)	17 (6.8 – 29.3)
2021	8	640	574 (524 – 624)	66 (16 – 116)	11.5 (2.6 – 22.1)
2021	9	843	629 (575 – 683)	214 (160 – 268)	34 (23.4 – 46.5)

Table I. Eating disorders

Year	Month	Observed	Predicted No.	Absolute difference No.	Relative difference %
2020	3	66	79 (66 – 91)	-13 (-25 – 0)	-16 (-27.7 – 0.1)
2020	4	85	70 (58 – 81)	15 (4 – 27)	22 (4.5 – 46.7)
2020	5	67	72 (60 – 84)	-5 (-17 – 7)	-7.3 (-20.5 – 11.2)
2020	6	62	69 (57 – 81)	-7 (-19 – 5)	-10 (-23 – 8.3)
2020	7	61	49 (40 – 59)	12 (2 – 21)	24.1 (4.1 – 53.5)
2020	8	74	68 (57 – 80)	6 (-6 – 17)	8.6 (-7.2 – 30.7)
2020	9	92	75 (63 – 88)	17 (4 – 29)	22.3 (5.1 – 46.2)
2020	10	96	80 (67 – 92)	16 (4 – 29)	20.4 (3.8 – 43.4)
2020	11	101	86 (73 – 99)	15 (2 – 28)	17.4 (1.5 – 39.1)
2020	12	123	67 (56 – 78)	56 (45 – 67)	83.5 (56.7 – 121.2)
2021	1	96	79 (66 – 92)	17 (4 – 30)	21.4 (4.6 – 44.6)
2021	2	94	67 (55 – 78)	27 (16 – 39)	40.9 (20.6 – 69.5)
2021	3	118	83 (68 – 99)	35 (19 – 50)	41.7 (19.1 – 74.8)
2021	4	94	74 (59 – 88)	20 (6 – 35)	27.4 (6.5 – 58.3)
2021	5	91	77 (62 – 91)	14 (0 – 29)	18.8 (-0.4 – 47.4)
2021	6	116	73 (59 – 87)	43 (29 – 57)	58.9 (32.8 – 97.6)
2021	7	64	52 (41 – 63)	12 (1 – 23)	22.8 (1 – 56.7)
2021	8	79	72 (58 – 86)	7 (-7 – 21)	9.4 (-8.6 – 36.1)
2021	9	105	80 (64 – 95)	25 (10 – 41)	31.7 (10.5 – 62.9)

Table J. Neurodevelopmental disorders

Year	Month	Observed	Predicted No.	Absolute difference No.	Relative difference %
2020	3	835	840 (792 – 889)	-5 (-54 – 43)	-0.6 (-6 – 5.5)
2020	4	746	829 (781 – 877)	-83 (-131 – -35)	-10 (-14.9 – -4.5)
2020	5	852	896 (845 – 947)	-44 (-95 – 7)	-4.9 (-10 – 0.8)
2020	6	830	748 (704 – 793)	82 (37 – 126)	10.9 (4.7 – 17.9)
2020	7	275	236 (215 – 257)	39 (18 – 60)	16.6 (7.2 – 27.7)
2020	8	697	816 (769 – 864)	-119 (-167 – -72)	-14.6 (-19.3 – -9.4)
2020	9	800	877 (827 – 927)	-77 (-127 – -27)	-8.8 (-13.7 – -3.2)
2020	10	805	873 (824 – 923)	-68 (-118 – -19)	-7.8 (-12.8 – -2.3)
2020	11	827	877 (827 – 927)	-50 (-100 – 0)	-5.7 (-10.8 – 0)
2020	12	784	584 (547 – 622)	200 (162 – 237)	34.2 (26.1 – 43.3)
2021	1	898	929 (877 – 981)	-31 (-83 – 21)	-3.3 (-8.5 – 2.4)
2021	2	1039	857 (808 – 906)	182 (133 – 231)	21.2 (14.6 – 28.5)
2021	3	1165	884 (825 – 944)	281 (221 – 340)	31.7 (23.4 – 41.3)
2021	4	1113	872 (813 – 931)	241 (182 – 300)	27.6 (19.5 – 36.8)
2021	5	1027	943 (880 – 1006)	84 (21 – 147)	8.9 (2.1 – 16.7)
2021	6	981	788 (733 – 842)	193 (139 – 248)	24.5 (16.5 – 33.8)
2021	7	268	248 (225 – 272)	20 (-4 – 43)	7.9 (-1.3 – 19.1)
2021	8	931	859 (801 – 918)	72 (13 – 130)	8.3 (1.5 – 16.2)
2021	9	1055	923 (861 – 985)	132 (70 – 194)	14.3 (7.1 – 22.5)

Table K. Conduct and oppositional disorders

Year	Month	Observed	Predicted No.	Absolute difference No.	Relative difference %
2020	3	145	152 (136 – 168)	-7 (-23 – 9)	-4.3 (-13.5 – 7)
2020	4	165	149 (134 – 165)	16 (0 – 31)	10.5 (-0.1 – 23.6)
2020	5	131	166 (149 – 183)	-35 (-52 – -18)	-21.1 (-28.4 – -12.1)
2020	6	126	132 (117 – 147)	-6 (-21 – 9)	-4.4 (-14 – 7.5)
2020	7	50	61 (52 – 70)	-11 (-20 – -2)	-18.3 (-29 – -3.8)
2020	8	122	127 (113 – 142)	-5 (-20 – 9)	-4.1 (-13.8 – 8)
2020	9	149	145 (130 – 161)	4 (-12 – 19)	2.7 (-7.2 – 15)
2020	10	165	155 (139 – 171)	10 (-6 – 26)	6.3 (-3.8 – 18.7)
2020	11	193	167 (150 – 184)	26 (9 – 43)	15.6 (4.9 – 28.8)
2020	12	115	110 (97 – 123)	5 (-8 – 18)	4.2 (-6.8 – 18.2)
2021	1	151	167 (149 – 184)	-16 (-33 – 2)	-9.3 (-17.8 – 1.1)
2021	2	147	146 (130 – 161)	1 (-14 – 17)	0.9 (-8.8 – 12.9)
2021	3	156	146 (128 – 164)	10 (-8 – 28)	7 (-4.8 – 22.1)
2021	4	126	144 (126 – 162)	-18 (-36 – 0)	-12.4 (-22 – 0.1)
2021	5	135	160 (140 – 179)	-25 (-44 – -5)	-15.5 (-24.6 – -3.9)
2021	6	122	127 (111 – 143)	-5 (-21 – 11)	-3.9 (-14.8 – 10.3)
2021	7	46	59 (49 – 69)	-13 (-23 – -3)	-21.9 (-32.9 – -6.6)
2021	8	100	122 (107 – 138)	-22 (-38 – -7)	-18.3 (-27.7 – -6.2)
2021	9	119	140 (122 – 157)	-21 (-38 – -3)	-14.8 (-24.3 – -2.6)

Table L. Self-harm

Year	Month	Observed	Predicted No.	Absolute difference No.	Relative difference %
2020	3	58	56 (44 – 67)	2 (-9 – 14)	4.3 (-14.1 – 32.8)
2020	4	50	67 (53 – 80)	-17 (-30 – -3)	-24.9 (-37.5 – -5.9)
2020	5	49	69 (55 – 82)	-20 (-33 – -6)	-28.6 (-40.5 – -10.7)
2020	6	44	48 (37 – 58)	-4 (-14 – 7)	-7.5 (-24.6 – 19.5)
2020	7	43	48 (37 – 59)	-5 (-16 – 6)	-10.4 (-26.9 – 15.7)
2020	8	53	68 (54 – 82)	-15 (-29 – -1)	-22.1 (-35.1 – -2.5)
2020	9	71	64 (51 – 77)	7 (-6 – 20)	11.5 (-7.4 – 40.3)
2020	10	50	70 (56 – 84)	-20 (-34 – -6)	-28.4 (-40.3 – -10.5)
2020	11	61	68 (54 – 81)	-7 (-20 – 7)	-9.9 (-25 – 12.8)
2020	12	49	62 (50 – 75)	-13 (-26 – -1)	-21.4 (-34.9 – -1)
2021	1	40	92 (75 – 109)	-52 (-69 – -35)	-56.4 (-63.2 – -46.3)
2021	2	43	58 (46 – 70)	-15 (-27 – -3)	-26.2 (-38.9 – -6.8)
2021	3	52	65 (49 – 80)	-13 (-28 – 3)	-19.5 (-35.4 – 6.9)
2021	4	47	77 (59 – 96)	-30 (-49 – -12)	-39.2 (-50.8 – -20.5)
2021	5	42	80 (61 – 98)	-38 (-56 – -19)	-47.3 (-57.3 – -31.1)
2021	6	35	55 (41 – 69)	-20 (-34 – -6)	-36.7 (-49.6 – -14.8)
2021	7	37	56 (41 – 70)	-19 (-33 – -4)	-33.6 (-47.1 – -10.7)
2021	8	38	79 (60 – 98)	-41 (-60 – -22)	-51.9 (-61.1 – -37.1)
2021	9	54	74 (56 – 92)	-20 (-38 – -2)	-27 (-41 – -4.1)
