# Supplementary material for: Comparison of new psychiatric diagnoses among Finnish children and adolescents before and during the COVID-19 pandemic: A nationwide register-based study
Source: PLoS Med. 2023 Feb 27;20(2):e1004072. doi: 10.1371/journal.pmed.1004072 (PMC10089356; doi:10.1371/journal.pmed.1004072)
Supplement: Attachment — Submitted filename: Response letter.docx [file pmed.1004072.s006.docx]

COVERING LETTER WITH DETAILED RESPONSES TO REVIEWERS’ COMMENTS

Manuscript reference number: PMEDICINE-D-22-01112R1

Dear Dr. Callam Davidson,

We wish to thank you and the reviewers for a thorough review. We were delighted to hear that reviewer #1 “thoroughly enjoyed reading the paper”, “this study adds significantly to the existing literature”, it is “well-conducted population study” and the “study has clear clinical implications”. We are also grateful for the critical issues that the reviewers raised to improve the manuscript. We have addressed all comments and revised our manuscript in light of the editor’s and reviewers' comments. Please find below our responses point-by-point; for convenience and possible cross-reference between comments, we have added numbering to the comments and renumbered comments when they included several suggestions.

If the manuscript is accepted for publication, we would like the manuscript to be considered for the special issue on the COVID-19 pandemic and global mental health.

Please do not hesitate to contact me if you would have any further inquiries or comments.

Best regards on the behalf of the authors,

David Gyllenberg

************************************************************

************************************************************

Requests from the editors:

Editor / comment #1

Please revise your title according to PLOS Medicine's style. Your title must be nondeclarative and not a question. It should begin with main concept if possible. "Effect of" should be used only if causality can be inferred, i.e., for an RCT. Please place the study design ("A randomized controlled trial," "A retrospective study," "A modelling study," etc.) in the subtitle (ie, after a colon).

Response: We have revised the title accordingly.

Edits: “Comparison of Population-based comparisons of new psychiatric diagnoses among Finnish children and adolescents before and during the COVID-19 pandemic: A nationwide register-based study”

***

Editor / comment #2

Please structure your abstract using the PLOS Medicine headings (Background, Methods and Findings, Conclusions).

Response: The abstract is structured accordingly using the PLOS Medicine headings

***

Editor / comment #3

Abstract Methods and Findings:

* Please quantify the main results with 95% CIs.
* Please include the important dependent variables that are adjusted for in the analyses.
* In the last sentence of the Abstract Methods and Findings section, please describe the main limitation(s) of the study's methodology.

Response: We have revised the abstract accordingly.

***

Editor / comment #4

Thank you for including an Author Summary. Please trim the summary such that bullet points are single sentence (maintaining 2-3 bullet points per question). Bullet points should be objective, brief, succinct, specific, accurate, and avoid technical language.

Response: We have trimmed bullet points in the author summary.

***

Editor / comment #5

Please include continuous line numbering throughout your manuscript document.

Response: We will add line numbering to the revised version of our submission.

Edits: Added line numbering to the revised manuscript

***

Editor / comment #6

Please place citations within square brackets and have them preceding punctuation. 

Response: We have modified all in-text citations as described.

Edits: Edited all in-text citations to aforementioned format.

***

Editor / comment #7

Please update your in-text citations for Supplementary Materials using the guidance found here: <https://journals.plos.org/plosmedicine/s/supporting-information#loc-item-description>

Response: We have modified all in-text citations to supplementary materials as described.

Edits: Changed “supplemental figure” to “S1 Figure” and named other supplementary materials accordingly

***

Editor / comment #8

Please ensure that the study is reported according to the STROBE guideline, and include the completed STROBE checklist as Supporting Information.  Please add the following statement, or similar, to the Methods: "This study is reported as per the Strengthening the Reporting of Observational Studies in Epidemiology (STROBE) guideline (S1 Checklist)."

The STROBE guideline can be found here: <http://www.equator-network.org/reporting-guidelines/strobe/>

When completing the checklist, please use section and paragraph numbers, rather than page numbers.

Response: We have added the STROBE checklist as S1 Checklist, and added the supplied statement to Methods.

***

Editor / comment #9

Did your study have a prospective protocol or analysis plan? Please state this (either way) early in the Methods section.

a) If a prospective analysis plan (from your funding proposal, IRB or other ethics committee submission, study protocol, or other planning document written before analyzing the data) was used in designing the study, please include the relevant prospectively written document with your revised manuscript as a Supporting Information file to be published alongside your study, and cite it in the Methods section. A legend for this file should be included at the end of your manuscript. 
b) If no such document exists, please make sure that the Methods section transparently describes when analyses were planned, and when/why any data-driven changes to analyses took place.  
c) In either case, changes in the analysis-- including those made in response to peer review comments-- should be identified as such in the Methods section of the paper, with rationale.

Response: We have added the analysis plan as S3 Supporting Information, and we have added text to the methods when deviation from the plan has been made.

***

Editor / comment #10

Typo on page 12: Figure A should read Figure 1A.

Response: We have corrected the typo.

***

Editor / comment #11

Typo in legend for Figure 1: September 202 should read September 2021.

Response: We have corrected the typo.

***

Editor / comment #12

Please check your y-axis labels are correct – I think they should all read ‘per 1,000’ as opposed to ‘for 1,000’.

Response: We have changed the label.

***

Editor / comment #13

I would suggest using a solid and a dashed line to differentiate between observed/predicted rates in your figures, as the black vs grey distinction is not always obvious (though inclusion of 95% CI does make this easier). 

Response: We have edited the figures accordingly.

***

Editor / comment #14

In the Results section, please quantify results with 95% CI. 

Response: We have quantified the results with 95% CI.

***

Editor / comment #15

Please ensure claims made in the Discussion are suitably referenced (e.g. ‘restrictions were more likely to affect the kind of behaviours that teenagers engaged in, like hanging out with friends’).

Response: We have ensured that the claims in the discussion are suitably referenced.

Edits: ”It is also possible that adolescents reacted to physical distancing measures more strongly than younger children. This is because the restrictions were more likely to affect their friendship dynamics [25] especially considering adolescence period is connected to increased salience on peer relationship [26].”

***

Editor / comment #16

The Author’s contributions, conflicts of interest statements, role of the funding source, and ethics committee approval, and data sharing statement sections can all be removed from the end of the main text (pages 21-22) – all of the information is captured elsewhere (either in the Submission form or the Methods). 

Response: We have now removed this text

************************************************************

************************************************************

Comments from the reviewers:

Reviewer #1:

R1 / comment #1

Please check grammar and spellings throughout. A native English speaker may be able to help. 

Response: We have checked the grammar and spelling throughout.

***

R1 / comment #2

Title: Appropriate

Abstract: 
Reflects the contents within the manuscript. 

Introduction:
Very well-written. Evidence before the study well-summarised and aims of the study easy to follow

Methods:
What was the rationale behind stratification by age?

Response: The rationale behind stratification by age is that, in Finland, child psychiatry and adolescent psychiatry are separate specialties, and the age groups 0–12 years versus 13–17 years are typically used to determine which specialty the patient belong to.

Edits: Outcomes: “In Finland, child psychiatry and adolescent psychiatry are separate specialties, and the age groups 0–12 years versus 13–17 years are typically used to determine which specialty the patient belongs to.”

***

R1 / comment #2

I note the authors include different diagnosis as separate incidents. Can the authors preclude a wrong first diagnosis initially? I.e. is there any other reason why we might be seeing a spike, such as historically wrongly diagnosed cased being correctly diagnosed due to advancements in knowledge/reclassification of diseases.
Overall, very detailed and replicable

Response: We thank the reviewer for the comment. We cannot determine from register-based data whether a patient’s first diagnosis is initially incorrect and later corrected, and we have now discussed this in the limitations section.

Edits: Limitations: “Fourth, unfortunately, we cannot from register-based data determine whether a patient’s first psychiatric diagnosis is initially incorrect and later corrected. While such a correction would not affect the main outcome ‘any diagnosis’, it could lead to an increased number of specific diagnoses. Nonetheless, we do not have reason to assume that the level of this such kind of a correction process would be increased during the COVID-19-pandemic and that it could explain the increased observed rates.”

***

R1 / comment #3

Results:
Given we are nearly half-way into 2022, is there still no data on the population at risk in 2021? I would recommend actual numbers replace estimated numbers where possible. 
I'm struggling to correlate the figures with the data presented in the tables. There seems to be a reduction in the number of diagnosis across Finland in 2021 compared to previous years (Table 1). I appreciate that this data is only until September, and this may be higher than values in September from previous years. Also, it may be that the under-diagnosis and over-diagnosis shown in the figures evened out long-term, but this doesn't reflect the results presented in Table 2. I'd like the data to be presented in way that is easier to correlate the table and figure findings. This can be done through legends if need be.

Response: We have updated the population at risk for 2021 with actual data in Table 1 and rerun all analyses with that data; no major changes were noted, except for frequencies and decimals shown in Table 1 and the results section. To easier correlate the findings in Table 2 and the figures, we have also added a light gray shaded area to represent the first wave months in all figures.

Edits: Please see Table 1 and Figures.

Discussion:
Very well written

I thoroughly enjoyed reading the paper.

************************************************************

************************************************************

Reviewer #2: The study by Gyllenberg et al. leverages nationwide Finnish data to address an extremely important topic on potential change in the incidence of psychiatric disorders among children (as diagnosed through the health care system) during the first 16 months of the COVID-19 pandemic.  With a clear pattern in the results, this study adds significantly to the existing literature. I hope the following points are helpful to the authors to improve the quality of their manuscript:   

***

R2 / comment #1

1.	The decrease in incident substance use disorders and self-harm from June 2020 to September 2021 among children is interesting and for a balanced review of the results these findings should also be discussed and commented on, e.g. in the abstract and sections of discussion/conclusion in the paper. 

Response: We thank the reviewer for the comment. We have now added a paragraph discussing these findings and reported them in the abstract.

Edits:

- Discussion: ”While we noted higher observed numbers of new diagnoses for several diagnostic groups than predicted during the months following the first phase of the pandemic, psychotic and bipolar disorders and conduct and oppositional disorders did not increase, and self-harm and substance use disorders decreased. The findings related to conduct and substance related disorders are in line with previous study showing that internalizing problems, such as depression and anxiety, have increased more among adolescents during the pandemic than out-acting problems [27]. The number of patients or visits with self-harm and suicidal behaviours have increased during the pandemic according to a study [28] or decreased according to a multinational study [14]. This study adds to the literature by showing that new diagnoses of hospital-presenting self-harm decreased in Finland during the pandemic. The finding of no changes in new diagnoses of psychotic or bipolar disorders in Finnish specialist services during early or later phases of the pandemic is reassuring, as it shows that help-seeking and diagnostics of these severe disorders does not seem to be affected by lockdowns.”
- Abstract: “The largest increases by diagnostic groups were found for eating disorders (27.4%, 8.0-55.3), depression and anxiety (21.0%, 12.1-51.9) and neurodevelopmental disorders (9.6%, 3.0 – 17.0), but psychotic and bipolar disorders and conduct and oppositional disorders showed no significant differences; and self-harm (-28.6, -41.5 - -8.2) and substance use disorders (-15.5, -26.4 - -0.7) decreased in this period.”

***

R2 / comment #2

2.	The first sentence of the introduction: „Psychiatric symptoms increased among children and adolescents after the start of the COVID-19 pandemic,1-3 but service use reduced, according to a systematic review" - needs to be clarified.  Was this a global thing or occurred in a particular setting? Also, psychiatric symptoms have in many societies been increasing in children, particularly teens, during the last 10 years.  I think clarifications are needed here.

Response: We agree with the reviewer that the sentence needs clarification. We primarily cite repeated cross-sectional studies, and clarify that these have been European studies. We have also added a sentence to the first paragraph of the introduction about trends before the onset of the pandemic.

Edits: Introduction: “Several studies in Europe showed mental health symptoms increased among children and adolescents after the start of the COVID-19 pandemic [1-3], but service use reduced, according to a systematic review [4]. Detailed information is vital if we are to mitigate the mental health consequences of the COVID-19 pandemic in these patients. This needs to include changes in the predicted versus observed use of psychiatric services and the demographic and diagnostic groups that are most affected by the pandemic. Because of increasing new psychiatric diagnoses already before the onset of the pandemic [5], these potential trends need to be taken into account.”

***

R2 / comment #3

3.	Introduction, second to last paragraph. The rationale for the study aims lacks mentioning of how limited health consumption data are in quantifying the „need" for psychiatric services during a pandemic, when populations are advised to stay at home.  Thus, estimation of incidence or new onset psychiatric disorders are limited by this factor.

Response: As suggested by Reviewer 2, an additional rationale has been added

Edits: Introduction: “Finally, there is limited data that quantifies the number of new diagnoses in psychiatric services during the COVID-19 pandemic, when different populations at different timepoints have been advised to stay at home.”

***

R2 / comment #4

4.	Introduction, specific aims/last paragraph: „The aim of this study was to compare the pandemic restrictions in Finland with the number of children and adolescents who received a new primary or secondary diagnoses from psychiatric specialized services."  The aim is not clearly stated, please consider to rephrase. The aim here must be to compare the incidence of primary and secondary psychiatric diagnoses in children across periods and places of varying pandemic restrictions?      

Response: We thank the review for pointing out the unclearness and the suggestion; we have used the suggestions with a minor modification. We have now rephrased the primary aim and clarified the other aims.

Edits: Introduction: ”The aim of this study was to compare the number of children and adolescents with new primary or secondary diagnoses across periods of varying pandemic restrictions” … ”Our secondary aim was to examine new diagnoses by sex, age group and geographic areas, as restrictions were different for children versus adolescents and in the capital and surrounding areas versus rest of Finland.”

***

R2 / comment #5

5.	I noticed a few typos and grammatic errors (e.g. page 8, line 2), as well as use of the English language where I am unsure that the correct meaning is conveyed (as in point 3 above) - please review carefully.

Response: We have corrected typos and grammatical errors throughout.

***

R2 / comment #6

6.	Methods, first paragraph: „The Finnish Institute for Health and Welfare granted us permission to use the data (decision number THL/1745/6.02.00/2021)."  What about ethics approval?  Was the study exempt from such a review?

Response: Thank you very much. We have updated the ethical approval process.

Edits: Methods: “The Finnish Institute for Health and Welfare (THL) granted us permission to use the data (decision number THL/1745/6.02.00/2021). The THL reviewed that the study plan is in line with the data permission and that data-security-related and other ethical issues are fulfilled. Therefore, a separate ethics approval was not needed.”

***

R2 / comment #7

7.	Methods, first paragraph: It is stated that the study used aggregated data while later (page 8, 2nd paragraph) it is stated: "The data we gathered included the personal identification number issued to all Finnish citizens and the primary and secondary diagnoses, using the International Classification of Diseases, Tenth Revision (ICD-10) codes."   Please clarify.

Response: We thank the reviewer for noticing this. We have now clarified the text. Please also see response to R5 / comment #12.

Edits: Methods: “The data used for the aggregated data included the personal identification number issued to all Finnish citizens and the primary and secondary diagnoses, using the International Classification of Diseases, Tenth Revision (ICD-10) codes [21].”

***

R2 / comment #8

8.	Methods (page 9, paragraph 1): „We tracked the data for each individual back to birth to make sure that we only recorded the first diagnosis for any specific disorder."  Was this conducted using the same data used during the study period (2017-2021)?  Any information on the quality of this data source or validation studies on psychiatric disorders?

Response: Yes, the study was conducted using the same data during the study period 2017-2021. The incidence of diagnoses was defined as any new outcome-specific primary or secondary diagnosis that a patient received during the study period. We have mentioned it in the method section. Additionally, we have included references to the quality of this data source [20] and validation studies [5] on psychiatric disorders.

Edits: Methods: “The incidence of diagnoses was defined as any new outcome-specific primary or secondary diagnosis that a patient received during the study period. We tracked the data for each individual back to birth to make sure that we only recorded the first diagnosis for any specific disorder. For example, if a patient received a diagnosis of depression in 2017 and a diagnosis of psychosis in 2020, the data relating to those specific diagnoses were included in those two years as two separate incidents.”

***

R2 / comment #9

9.	Methods/statistical analysis (page 9-10):  The description is quite general and would benefit for more details on the approach/modeling, subgroup analyses and rationale for these decisions.

Response: We have now added more details on the approach, sub-group analyses and rationale for the decisions.

Edits: Methods:

- ”We used negative binomial regression models appropriate for fitting count data and prediction of two-sided errors.”
- ”After conducting these analyses for the main outcome ‘any psychiatric diagnosis’ in the total data, we conducted sub-analyses of any diagnosis stratified by sex, age group and geographic area to examine whether findings were consistent in males or females or in groups differentially affected by restrictions. Finally, we conducted sub-analyses for diagnostic groups to examine whether the number of new diagnoses of particular types of disorders changed during the pandemic.”
- ”When plotting the monthly observed and predicted rates, we used rates instead of counts for increased interpretability and comparison across analyses.”

***

R2 / comment #10

10.	In a surge of a new somatic disease during a pandemic, you may hypothesize a similar surge in secondary diagnoses of anxiety and depression in this patient population. In Tables 1 and 2, it would therefore be interesting to show numbers of primary and secondary diagnoses, and preferably conduct an additional analysis contrasting expected and observed numbers by primary and secondary diagnoses.

Response: We agree with the reviewer that this would be an interesting topic. However, we do not have these data at hand, and retrieving data for additional analyses would take time. As it is not the primary aim of the study, we suggest to not proceed with the analyses.

************************************************************

************************************************************

Reviewer #3: This is a well-conducted population study on new psychiatric diagnoses among children and adolescents before and during the COVID-19 pandemic in Finland. The study design, datasets, statistical methods and analyses, and presentation (tables and figures) and interpretation of the results are mostly adequate and of a good standard. However, there are still a couple of issues needing attention.

***

R3 / comment #1

1) The use of negative binomial regression models is appropriate and the results are mostly descriptive in terms of diagnosis rates, trends/patterns and changes before and during Covid19 pandemic. We can see the patterns of rates are very consistent in the 3 years before pandemic (2017, 2018 and 2019) so we would expect them to be the same if Covid19 never happened. The study results showed that there is a drop (not statistically significant) in the first 3 months of the pandemic and then the rates picked up later (18.4%). Then the authors concluded "This increase can probably be explained by changes in helpseeking, referrals and psychiatric problems, rather than delays in accessing services" (Page 20, conclusion). However, I am not convinced with this statement. They could all be factors for the diagnosis rate changes during the pandemic. So far the paper is mostly descriptive rather than multivariable analysis on factors contributing to the rate changes. One would imagine
the delays in hospital service during the pandemic would be the most important factors of this rate change or at least a very important factor. Could authors provide the hospital service data before and during the pandemic such as consultancy appointments, closures, reduced staff capacity (%?), policy changes in hospital services, and etc? We need some solid data to support the claims of factors leading to these rate changes so that we can understand better and be prepared if it happens again.

Response: We appreciate that the reviewer raised these important issues. We have now revised the conclusion about delays in accessing services and clarified what kind of conclusions can be made from nationwide data with follow-up from birth. Unfortunately, we do no not have access to quantitative data on changes in staff capacity, waiting times and policy changes across service systems. We recognize this as a limitation and discuss this in the manuscript. Please also see response to R4 / comment #3.

Edits:

- Abstract conclusions: “Possible explanations to our findings include changes in help-seeking, referrals and psychiatric problems and delayed service access.”
- 1st paragraph in discussion: “The finding that new diagnoses were a fifth higher than our predictions, after the first phase of the COVID-19 pandemic in spring 2020, can probably be explained by changes in help-seeking, delayed treatment, referrals and psychiatric problems.”
- New paragraph in discussion: “Plausible explanations of the increase in new diagnoses include delayed access to services, changes in help-seeking, referrals and psychiatric problems… “
- Limitations: “Third, while the nationwide data with follow-up from birth enabled us to study the specialist service system’s actualized capacity to diagnose new cases in times of changed demand, we did not have quantitative data on changes in staff capacity, waiting times and policy changes across service systems. Multivariable time series studies with more information on these factors would be important for deepened understanding of the changes and for being prepared for new pandemics.”
- Conclusion: “This increase is likely explained by changes in help-seeking, referrals and psychiatric problems and delayed access to services.”

***

R3 / comment #2

2) The authors haven't mentioned anything about missing data. What's the quality of the data? Any missing data issues?

Response: We have now added a sentence about missing data. We have also added citations about the quality about the data. Please see repsonse to R2 / comment #8.

Edits: ”We retrieved the aggregated data on 26 November 2021, but we excluded the data from October 2021 from the final analyses, as it was incomplete for certain geographic areas. The final included data was considered complete and full case analyses were conducted.”

************************************************************

************************************************************

Reviewer #4: 

The current study examined the prevalence of new primary and secondary diagnoses from psychiatric specialized services among Finnish children and adolescents in the wake of the COVID-19 pandemic. The differences in prevalence rates were compared between when restrictions rates were the tightest (March-May 2020) and once the restrictions were eased (June 2020-September 2021) to pre-pandemic prevalence rates (January 2017-February 2020). They found a decrease in mental health services used during the first three months of the pandemic, which was followed by an increase in the 16-months that followed. The study has clear clinical implications regarding the mental health status of youth during the pandemic and the need for further resources to be devoted to prevention and intervention efforts for this vulnerable group.

***

R4 / comment #1

There is substantial research indicating that the rates of depression, anxiety and self-harm have been on the rise among adolescents prior to the pandemic. As such, there may be an upward trend in these diagnosis during the 2017-2020 period. Was the increase in depression and anxiety after the first wave of the COVID-19 pandemic greater than this trend would suggest? The answer is likely yes; however, looking into this could provide more insight into how much the pandemic and its restrictions have negatively impacted the mental health of adolescents. 

Response: We thank the reviewer for pointing this out. Yes, an increase in depression and anxiety diagnoses after the first wave of the COVID-19 pandemic was greater than previous trends would suggest and we took this into account in the analyses. We have added text to the discussion clarifying this.

Edits:

- Introduction, first paragraph, last sentence: “Because of increasing new psychiatric diagnoses already before the onset of the pandemic [5], these potential trends need to be taken into account.”
- Discussion, strengths and limitations paragraph: “The predicted number of diagnoses took into account possible previous increasing trends and seasonal variation, meaning that differences between observed and predicted diagnoses cannot be explained by for example previous rising service use [5].”

***

R4 / comment #2

Whether the prevalence rate increases or decreases varies considerably by disorder. If space allows, it would be interesting to provide more information on why these rates differ. For instance, any thoughts on why prevalence rates on self-harm decreased after the pandemic? Or why the prevalence of eating disorders increased?

Response: We agree with the reviewer that the differing findings for different disorders are interesting. We have now added more discussion about disorder-specific findings – also the incidence rates that decreased (R2 / comment #1) – and how the findings relate to the literature. To keep the discussion compact, we suggest not including too much text about possible reasons why there are different changes for different disorders.

Edits: Please see response to R2 / comment #1

***

R4 / comment #3

In the discussion, the authors note that the increase in new diagnosis after the first phase of COVID-19 is likely due to changes in help-seeking, referrals and psychiatric problems rather than delayed access to services due to the pandemic restrictions. On what do the authors base this claim?

Response: This issue was also raised by another reviewer (R3 / comment #1) and we have now revised this and added a new paragraph to the discussion section on the subject.

Edits: Discussion: “Plausible explanations of the increase in new diagnoses include delayed access to services, changes in help-seeking, referrals and psychiatric problems. A WHO survey conducted in 130 countries between June and August 2020 showed that 72% countries reported disruption in services for children and adolescents with mental, neurological and substance use disorders during the COVID-19 pandemic [22]. Access to care may have been disrupted due to the fear of infection of parents and caregivers causing delayed access to services [23]. This changed help-seeking and delay in treatment could have been one reason to worsening of symptoms that later in the pandemic required referral to specialist services. For example, referrals to child and adolescent mental health services recorded an initial decrease during the early phase of the pandemic followed by substantial increase as the pandemic progressed in the Republic of Ireland and the UK [10,24]. Evidence for increased symptoms has for example been provided by two ongoing trials in the UK: They found that the prevalence of adolescents with high depressive symptoms would have been 25% instead of 27% if the COVID-19 had not occurred [3].”

************************************************************

************************************************************

Reviewer #5: * The research question is an important one to the community of researchers in this general area.
Yes

* The results provide a substantial advance over existing knowledge, with clear implications for patient care, public policy, or clinical research agendas.
No

* Published together with an Author Summary written for general readers, the article is of interest to clinicians and policymakers who are not specialists in this topic.
Yes

* What are the main claims of the paper and how significant are they for the discipline?
The study confirms an increase in specific psychiatric diagnostic rates in adolescents starting a few months after the COVID pandemic. The study includes only first time diagnoses to determine how the pandemic is associated with mental health disorder incidence. It includes a longer time period than other studies (up until September 2021 instead of March 2021). Since these two approaches appear to be the main contributions, the findings are not particularly novel, at least compared to results from other countries. But it is beneficial to have country-specific results available, especially from those using nationally representative data with records going back to birth to determine a newly diagnosed disorder. And it is helpful to continue highlighting the importance of the associations between shocks like the COVID pandemic with mental health care utilization in youth.

*Are the claims properly placed in the context of the previous literature? Have the authors treated the literature fairly?
Yes

*Do the data and analyses fully support the claims? If not, what other evidence is required?
Yes

*PLOS Medicine encourages authors to publish detailed methods as supporting information online. Do any particular methods used in the manuscript warrant such publication? If a protocol is already provided, for example for a randomized controlled trial, are there any important deviations from it? If so, have the authors explained adequately why the deviations occurred?
N/A

*Is this paper outstanding in its discipline? If yes, what makes it outstanding? If not, why not?
No. The use of a national registry is beneficial when drawing conclusions. Besides specifically focusing on newly diagnosed disorders, the study is not particularly novel in either its methodology or findings.

*Does the study conform to any relevant guidelines such as CONSORT, MIAME, QUORUM, STROBE, and the Fort Lauderdale agreement?
Yes

*Are details of the methodology sufficient to allow the experiments to be reproduced?
Yes

*Is any software created by the authors freely available?
N/A

*Is the manuscript well organized and written clearly enough to be accessible to non-specialists?
Yes

***

R5 / comment #1

Abstract: 
"Changes in help-seeking, referrals and psychiatric problems are likely explanations for our findings." Versus? In the discussion it becomes clear that the other reason considered is the delay of services. This might be added to make it a less obvious/empty statement at first sight. Also, something could be added along the lines as "Considering the magnitude of excess diagnoses, changes in help-seeking, referrals and psychiatric problems are likely explanations for our findings, versus delayed access to services" 

Response: We thank Reviewer 5 for this feedback. The issue about delayed access to treatment was also raised by R3 in comment #1 and by R4 in comment #3. We have now revised these conclusions and discussed them with more nuance as described in the responses to the above-mentioned comments.

***

R5 / comment #2

What does specialized services mean in Finland? Do the authors mean to say specialist services? Please be explicit in what kind of services this includes, e.g. outpatient, emergency room, hospitalizations, what kind of mental health care providers (e.g. psychiatrists, psychologists, social workers, etc). Possibly in the abstract but at the very least in the main body of the text. 

Response: We thank the reviewer for pointing out the unclearness. Both the terms specialized services and specialist services have been used to describe the same thing, and we have now revised it to specialist services throughout the manuscript. In addition, we have added text describing the service system. Due to word count limits, we suggest to not include this information in the abstract.

Edits:

- Data sources: “The Register contains information on all patients admitted for specialist inpatient healthcare since 1969 and public outpatient visits since 1998 [5,20]. In Finland, public healthcare is divided into primary care and specialist services that compromise inpatient units, emergency rooms in conjunction with hospitals and outpatient units of various medical specialties. In psychiatry units, the providers are a variety of occupations including physicians, psychologists and nurses, but diagnoses are set by physicians.”
- Outcomes and stratifying variable: “We included visits from all medical specialties where the diagnoses had been given.”

***

R5 / comment #3

Introduction: 
Comment on sentence: "However, it does not describe the number of children and adolescents who are using services for the first time, for a new psychiatric disorder". Does this sentence only apply to the study that is referred to, or all other prior studies? In the section "Why was this study done?", it is mentioned that none of the prior studies study new diagnoses. As this is one of the few potentially unique perspectives of the current study (besides the longer follow up period), this might be good to repeat in the introduction, not only related to that one study. 

Response: The sentence applies to that particular previous study from Finland. We have updated the reference to make it clearer. We have also included this unique perspective in the introduction section. In addition, we have revised our statement in the section “Why was this done?” as “Previous studies compared limited pandemic and pre-pandemic periods and they did not cover the whole spectrum of psychiatric or neurodevelopmental disorders.”

Edits: Introduction: “The COVID-19 pandemic has potentially changed how people access services and what services they need. That is why it is important to quantify how many new paediatric patients require psychiatric services. Finally, there is limited data that quantifies the number of new diagnoses in psychiatric services during the COVID-19 pandemic, when different populations at different timepoints have been advised to stay at home.”

***

R5 / comment #4

Missing "is" in: "That is why it important to quantify how many new paediatric patients require psychiatric services"

Response: Updated as suggested.

***

R5 / comment #5

The sentence "The aim of this study was to compare the pandemic restrictions in Finland with the number of children and adolescents who received a new primary or secondary diagnoses from psychiatric specialized services" does not make a lot of sense - comparing restrictions with number of people. Please rephrase. 

Response: We thank the reviewer for pointing out the unclearness. We have now revised the aims as described in R2 / comment #4.

Edits: Please see R2 / comment #4.

***

R5 / comment #6

The introduction is thorough in its description of the prior studies, but I'm missing one CDC report that shows similar results (especially for eating disorders) in the US that the authors may wish to include (<https://www.cdc.gov/mmwr/volumes/71/wr/pdfs/mm7108e2-H.pdf>). 

Response: Thank you very much. We have included the above-mentioned study in the introduction.

Edits: Introduction: “Another study from the United States reported that the proportion of weekly emergency department visits among adolescent (12-17 years) females doubled for eating disorders during 2020-2022 compared to pre-pandemic period [8].”

***

R5 / comment #7

Methods:
Is the age range 0-17 years inclusive, up to kids' 18th birthday?

Response: The age range does not include the 18th birthday. We have clarified the text.

Edits: Study design, setting and time periods: “comprised children and adolescents when they were aged 0–17 years”

***

R5 / comment #8

The sentence: "and most children moved to online teaching" implies that the children were teaching the class. An alternative would be to use the words "online learning" or something similar.

Response: We thank the reviewer for the suggestion and we have now revised as suggested.

***

R5 / comment #9

In the US we would use the word "essential workers" instead of "vital key workers", but this might be different in Finland/Europe. 

Response: We thank the reviewer for the suggestion and we have now revised as suggested.

***

R5 / comment #10

"In early May 2020, these restrictive measures started to be gradual reduced" -> I would suggest "Starting in early May 2020, these restrictive measures were gradually reduced"

Response: We thank the reviewer for the suggestion and we have now revised as suggested

***

R5 / comment #11

What does "at risk" mean? Is that the population without any prior diagnoses of mental health disorders? Or without a prior diagnosis for a specific disorder? Please define at first mention. 

Response: We have now added clarification at first mention that we did not make any restriction or exclude anyone from the at-risk population. We also acknowledge this in the limitations section (please also see R5 / comment #14).

We acknowledge that if the primary aim had been to estimate the overall incidence rate for diagnoses and if it would be important to rule out any underestimation, the population at risk would ideally be defined separately in each analysis as the population without a prior diagnosis of the studied outcome. However, the main aim in this study was study changes and the changes are studied as counts, not rates, and the rates (that utilize the at population at risk) are shown for descriptive purposes. Therefore, we have defined the denominator, or the population at risk, without restrictions.

Edits:

- Data sources: “no restrictions to the definition of the at-risk population were made [19].”
- Limitations: “Finally, we used census data to determine the population at risk without taking into account emigration, immigration and prior diagnoses.”

***

R5 / comment #12

Did the authors identify the cases in the Care Register for Health Care themselves? If so, the data needed to reproduce the results are (presumably and rightfully) not available to readers. But that means that the authors are not fulfilling the obligation to make all data fully available. 

Response: Yes, we identified the cases in the Care Register for Health Care ourselves; we then used aggregated for all analyses. It is correct that individual-level nationwide data cannot be freely available. According to our data approval, we can make aggregated data available given that it does not include counts lower than five due to risk of identification. We have now revised the methods text so it is clear that we retrieved the aggregated data, and we have revised the journal-specific section about data availability.

Edits:

- Data sources: “We retrieved the aggregated data on 26 November 2021…”

***

R5 / comment #13

When taking together the specific diagnostic categories, does that equal the "any disorders" category, or are there diagnoses that do not fall in any of the categories? 

Response: We decided to not study all specific diagnostic categories, e.g. personality disorders, that belong to the any disorder category, because they were uncommon in this age group.

Edits: Outcome and stratifying variables: “We did not separately study, e.g., new diagnoses of personality disorders (F60-F69), due to low incidence in this age group.”

***

R5 / comment #14

Are people included if they weren't born in Finland? If so, how is determined whether they were diagnosed for the first time? Conversely, if people were born in Finland but left at some point (also temporarily), are they removed from the denominator? In other words, how do the authors deal with immigration and emigration? 

Response: We have now added text to the limitations section about immigration and emigration. Please also see R5 / comment #11.

Edits:

- Discussion, limitations: “Finally, we used census data to determine the population at risk without taking into account emigration, immigration and prior diagnoses. However, these proportions are small in comparison to the total population: for example, the yearly immigration rate was 0.7% among 0–19-year-olds and has remained stable [19]. Therefore, it is unlikely that they would affect the results of changes in new diagnoses.”
- Table showing the yearly emigration and immigration rates among 0-19-year-olds in Finland: https://github.com/davgyl/covid_psyserv

***

R5 / comment #15

Is it possible to be diagnosed/treated for mental health disorders outside of the system, in a way that it wouldn't show up in the register, e.g. in private practices?

Response: We thank the reviewer for opening this topic. It is possible to be diagnosed outside specialist services. We have now added text related to this.

Edits: Limitations: “Children and adolescents can also be diagnosed in primary care, and to a lesser extent in private care, and are accepted into specialist services upon referral if the disorder is severe. By focusing on specialist services, we were able to utilize nationwide data dating back to birth of the study subjects and thereby determine new diagnoses. However, more detailed data on diagnoses in various settings is needed to further deepen the understanding of help-seeking and referral practices.”

***

R5 / comment #16

Am I reading it correctly that if someone receives a diagnosis of depression first and bipolar disorder later, this is counted as two separate incidents, but if this is depression first and anxiety later this would not count as two incidents? What is the justification for the combinations of the diagnostic categories? In the same vein, could an increase in incidence also mean that there are more differential diagnoses? 

Response: The combination of diagnostic categories was based on prior work (Gyllenberg et al., Lancet Psychiatry) and we considered this a way to provide an easily interpretable overview on most diagnostic categories. We have now added text related to this in the methods section. The rationale to pool psychotic and bipolar disorders was to study these severe and rather rare disorders at this age in the same group. Depressive and anxiety disorders were pooled because they are highly comorbid and common. With regard to the question about differential diagnosis, please see response to R1 / comment #2.

Edits: Outcomes and stratifying variables: ”… in line with a previous report [20] and to provide an easily interpretable overview on most diagnostic categories ”

***

R5 / comment #17

It is common to use a second event with a similar diagnosis to confirm that the disorder is present, and not just one event. At least for outpatient events or emergency room visits. This could prevent incorrect outcomes based on suspected cases, disorders that need to be ruled out, etc. How do the authors currently prevent this kind of error?  

Response: We agree with the reviewer that this would be a good strategy if it would be very high priority to avoid false positive diagnoses in the time series. Given that the aim is to study changes in registered diagnoses and we could not come up with an evident reason to believe that possible false positives would change over time, we opted to use one event.

***

R5 / comment #18

The phrase "the number with outcomes" is not clear - please specify which outcomes. 

Response: The reviewer is correct. We have now specified the outcomes.

Edits: Statistical analysis: ”… the number with any diagnosis and specific diagnostic groups …”

***

R5 / comment #19

Why did the authors not opt for methodology such as the Farrington algorithm (or adaptations) to detect whether the trends are different than the years before? Not necessarily a critique, mainly curious how these two approaches compare. I'd like to see some justification in the manuscript why the chosen approach is the most appropriate one though. 

Response: We thank the reviewer for the question. We looked into the Farrington algorithm during the review process. There is an R-package called ‘surveillance’ for implementing the Farrington algorithm to time series count data (<https://doi.org/10.18637/jss.v070.i10>). However, in its main implementation, it focuses on detecting (infectious disease) outbreaks i.e. observed counts over the upper bound, while our aim was to obtain both upper and lower limits of prediction and then compare to observed counts. We have added text related to this. Please also response to R2 / comment #9.

Edits: Statistical analysis lines: ”We used negative binomial regression models appropriate for fitting count data and prediction of two-sided errors”

***

R5 / comment #20

The sentence "Statistical power was defined as the 95% CI of the predicted numbers." is an odd interpretation of statistical power and/or CIs. Please explain and rephrase. 

Response: As this can be interpreted as posthoc statistical power, we suggest to omit this sentence.

Edits: Sentence omitted.

***

R5 / comment #21

Results
In the sentence "We stratified the analyses of any disorder by sex (male versus female), age group (0-12 versus 13-17 years) and geographic area (Helsinki University Hospital catchment area versus the rest of Finland)", "and" should be replaced with "or", as the cases were not stratified by all conditions at the same time. 

Response: The reviewer is correct.

Edits: “group (0-12 versus 13-17 years) or geographic area”

***

R5 / comment #22

Related to that, it would be interesting to see results from diagnostic categories by gender, sample size permitting. The CDC report mentioned earlier, with results from the US, shows increases in ED visits for eating disorders and tics only for girls and not boys. I wonder if that's the case here too, for different diagnostic categories.  

Response: We agree with the reviewer that this would be interesting. We have now conducted additional posthoc analyses related to outcomes that had sufficient sample size. Unfortunately, the low number of males with eating disorders did not allow for sex-stratified analyses.

Edits:

- Statistical analyses: “In posthoc supplemental analyses, we stratified analyses by sex for outcomes that had no frequencies under five in the sex-specific time series.”
- Results: “In additional sex-stratified analyses for selected outcomes, patterns in males and females were similar except for depression and anxiety where females had increased observed rates but males did not (S4 Figure).”

***

R5 / comment #23

Discussion
"During the first phase of the pandemic, we observed an 8% reduction in diagnoses for adolescents and a non-significant overall differences in new diagnoses". The second part of this sentence after "and" is not clear, with respect to describing a non-significant difference (use the phrase "no difference" instead, and use singular word not plural) and what outcome is described - does "overall" here mean 0-18 years since it is juxtaposed to adolescents? Please specify. 

Response: We thank the reviewer for detecting the unclear sentence. We have now corrected it.

Edits: Discussion: “During the first phase of the pandemic, we observed an 8% reduction in diagnoses for adolescents and no difference in new diagnoses in the total data.”

***

R5 / comment #24

Add "in" to the sentence "Another possible explanation for the increase diagnoses in adolescents and females is that…". 

Response: Changes done as requested.

***

R5 / comment #25

The sentence "These findings were in line with several reviews, which reported that older adolescents and females had depression and anxiety during the pandemic." doesn't make sense. Higher rates? 

Response: We thank Reviewer 5 for pointing this. We have revised the sentence accordingly.

Edits: Discussion: “These findings were in line with a meta-analysis study [6] which reported that older adolescents and females had higher rates of depression and anxiety during the pandemic.”

***

R5 / comment #26

Acknowledgements
Conceptulised -> conceptualised

Response: The ”Authors’ contribution” section has been removed from the main text, as noted in Editor / comment #16. The grammar has been corrected elsewhere

Edits: Omitted from text.
