# Supplementary material for: Comparison of new psychiatric diagnoses among Finnish children and adolescents before and during the COVID-19 pandemic: A nationwide register-based study
Source: PLoS Med. 2023 Feb 27;20(2):e1004072. doi: 10.1371/journal.pmed.1004072 (PMC10089356; doi:10.1371/journal.pmed.1004072)
Supplement: Attachment — Submitted filename: Response letter.docx [file pmed.1004072.s007.docx]

COVERING LETTER WITH DETAILED RESPONSES TO REVIEWERS’ COMMENTS

Manuscript reference number: PMEDICINE-D-22-01112R1

Dear Dr. Callam Davidson,

We are very happy to hear that “provided the remaining editorial and production issues are dealt with we are planning to accept the paper for publication in the journal”.

We have responded to the issues below.

Please do not hesitate to contact me if you would have any further inquiries or comments.

Best regards on the behalf of the authors,

David Gyllenberg

************************************************************

************************************************************

Requests from Editors:

Data Availability Statement: "Individual-level nationwide data cannot be made freely available in line with the data approval." Please provide more information regarding the data approval (e.g., that it was granted by the Finnish Institute for Health and Welfare/Decision number).

Response: The data availability statement has been updated as suggested. We have also made a DOI for the available data and code used for the analyses (doi: 10.5281/zenodo.6803574).

Edits: “Aggregated data used in the analyses are made freely available at https://github.com/davgyl/covid_psyserv (doi: 10.5281/zenodo.6803574). Individual-level nationwide data cannot be made freely available in line with the data approval granted by the Finnish Institute for Health and Welfare (decision number THL/1745/6.02.00/2021).”

***

Abstract: Please combine the Methods and Findings sections into one section, “Methods and findings”. The sentence detailing study limitations should come at the end of the 'Methods and Findings' section.

Response: This has been edited.

***

Author Summary: Please include include the headline numbers from the study, such as the sample size and key findings.

Response: The author summary has been edited accordingly.

Edits:

- “among approximately 1 million children and adolescents per year”
- “new diagnoses increased by 18.5%”

***

STROBE checklist: Please use section and paragraph numbers, rather than page numbers (as these are likely to be different in the published version).

Response: We have updated the checklist.

***

S2 Figure: Please update the y-axis label to read 'per 1,000' rather than 'for 1,000'.

Response: The y-axis is updated.

***

Figures 2 and 3: Please ensure that the y axis is identical for all panels to facilitate comparison.

Response: We have updated Figure 2 so that the y-axis is identical for all panels. For Figure 3, we suggest keeping the scale of the y-axis different across panels. This allows for comparison between observed and predicted rates within diagnostic groups – both rare and more common diagnostic groups. For example, if the scale for all panels in Figure 3 was to be set for 0–1.0 that includes all values for Depression and anxiety disorder, the observed and predicted rates for eating disorders and some other diagnostic groups would be very hard to compare. Instead, we suggest adding text the legend of Figure 3 that notes the differing scales.

Edits:

- Figure 2: The y-axis is identical.
- Legend of Figure 3: “The scale of the y-axis differs for the panels to facilitate comparison between observed and predicted rates within diagnostic groups.”

***

Please include a Table of data by month (similar to Table 2 but providing more granular insight by time). This can be included in the Supporting Information if preferred. 

Response: We have added tables by month in S4 Supporting information.

Edits:

- Results: “For more granular insight by time, the monthly numbers are described in S4 Supporting information.”
- S4 Supporting information

***

Discussion: Please expand on your interpretation of neurodevelopmental compared to psychiatric diagnoses; you note an increase in neurodevelopmental disorder diagnoses (beginning in December 2020) but this is not sufficiently discussed. 

Response: We agree. We have expanded our interpretation.

Edits: Discussion: “The new diagnoses of eating disorders and depression and anxiety disorders started to increase in September 2020 and those of neurodevelopmental disorders a few month later in December 2020. The later diagnoses of neurodevelopmental disorders might be explained by that more extensive assessments are needed for neurodevelopmental diagnoses.”

***

Comments from Reviewers:

Reviewer #2: The authors have addressed most of my points, and provided satisfactory explanations if not possible to address.
I have no further comments. 

Response: Thank you for reviewing the manuscript.

***

Reviewer #3: Thanks authors for their effort to improve the manuscript. I am satisfied with the response and revision. No further issues needing attention.

Response: Thank you for reviewing the manuscript.

***

Reviewer #4: Thank you for the opportunity to review this interesting and important study. The authors should be commended on their willingness and ability to incorporate the feedback from reviewers. Here below are a few minor issues for the authors to consider.

How reliable are diagnoses in the registry databases? Who assigns the diagnoses in outpatient settings (child and adolescents psychiatrists, clinical psychologists, general physicians?) and what methods are typically used to derive at a diagnosis (e.g., clinical interview vs. structured clinical interview)? The diagnoses in the current study are also based on ICD-10, not the more recent ICD-11. These might be points to reflect on in the limitations section.

Response: We thank the reviewer for the suggestion. We have elaborated on the validity of diagnoses in the methods section and added text about structured clinical interviews in the limitations section. Based on our clinical practice, structured clinical interviews, e.g. the K-SADS, is used in many clinics. However, as we lack nationwide data on these practices, we suggest to not elaborate on this in the manuscript.

Edits:

- Methods, Data sources: “diagnoses are set by child and adolescent psychiatrists or resident physicians. The diagnostic validity of schizophrenia [21], type I bipolar disorder [22], autism [23], ADHD [24] and Tourette’s syndrome [25] in the register has been studied.”
- Discussion, limitations: “However, more detailed data on diagnoses in various settings, preferably using structured clinical interviews with latest diagnostic criteria, is needed for better understanding help-seeking and referral practices.”
- References:
- 21. Mäkikyrö T, Isohanni M, Moring J, Hakko H, Hovatta I, Lönnqvist J. Accuracy of register-based schizophrenia diagnoses in a genetic study. Eur Psychiatry. 1998;13(2):57-62.
- 22. Kieseppä T, Partonen T, Kaprio J, Lönnqvist J. Accuracy of register- and record-based bipolar I disorder diagnoses in Finland; a study of twins. Acta Neuropsychiatr. 2000 Sep;12(3):106-9.
- 23. Lampi KM, Sourander A, Gissler M, Niemelä S, Rehnström K, Pulkkinen E, Peltonen L, Von Wendt L. Brief report: validity of Finnish registry-based diagnoses of autism with the ADI-R. Acta Paediatr. 2010 Sep;99(9):1425-8.
- 24. Joelsson P, Chudal R, Gyllenberg D, Kesti AK, Hinkka-Yli-Salomäki S, Virtanen JP, Huttunen J, Ristkari T, Parkkola K, Gissler M, Sourander A. Demographic Characteristics and Psychiatric Comorbidity of Children and Adolescents Diagnosed with ADHD in Specialized Healthcare. Child Psychiatry Hum Dev. 2016 Aug;47(4):574-82.
- 25. Leivonen S, Voutilainen A, Hinkka-Yli-Salomäki S, Timonen-Soivio L, Chudal R, Gissler M, Huttunen J, Sourander A. A nationwide register study of the characteristics, incidence and validity of diagnosed Tourette syndrome and other tic disorders. Acta Paediatr. 2014 Sep;103(9):984-90.

***

In the first sentence of the abstract, it is noted that there was a decrease in specialist psychiatric services for youth during the ´first phase of the pandemic´. It might be unclear to the reader what constitutes the ´first phase´. The authors should consider rewording here to clarify.

Response: We agree, and we have reworded it.

Edits: “COVID-19 restrictions decreased the use of specialist psychiatric services for children and adolescents in spring 2020.”

***

I believe there is a typo in line 274 where ´phrase´ is meant to be ´phase´.

Response: Thank you for noting the typo. We have corrected it.

***

Reviewer #5: The authors have addressed my main concerns and questions. However, during the revisions, more sentences with missing words, spelling/grammar errors, and uncommon sentence structures were introduced, and the authors need to receive input from one or more native English speakers.

Response: Thank you for the review. We have proofread the text again, corrected errors and changed sentence structures.
